# Age of avatar modulates the altercentric bias in a visual perspective-taking task: ERP and behavioral evidence

**DOI:** 10.3758/s13415-018-0641-1

**Published:** 2018-09-21

**Authors:** Heather J. Ferguson, Victoria E. A. Brunsdon, Elisabeth E. F. Bradford

**Affiliations:** School of Psychology, Keynes College, University of Kent, Canterbury, CT2 7NP UK

**Keywords:** Theory of Mind, Visual perspective-taking, Altercentric interference, Self/other, ERPs

## Abstract

Despite being able to rapidly and accurately infer their own and other peoples’ visual perspectives, healthy adults experience difficulty ignoring the irrelevant perspective when the two perspectives are in conflict; they experience egocentric and altercentric interference. We examine for the first time how the age of an observed person (adult vs. child avatar) influences adults’ visual perspective-taking, particularly the degree to which they experience interference from their own or the other person’s perspective. Participants completed the avatar visual perspective-taking task, in which they verified the number of discs in a visual scene according to either their own or an on-screen avatar’s perspective (Experiments 1 and 2) or only from their own perspective (Experiment 3), where the two perspectives could be consistent or in conflict. Age of avatar was manipulated between (Experiment 1) or within (Experiments 2 and 3) participants, and interference was assessed using behavioral (Experiments 1–3) and ERP (Experiment 1) measures. Results revealed that altercentric interference is reduced or eliminated when a child avatar was present, suggesting that adults do not automatically compute a child avatar’s perspective. We attribute this pattern to either enhanced visual processing for own-age others or an inference on reduced mental awareness in younger children. The findings argue against a purely attentional basis for the altercentric effect, and instead support an account where *both* mentalising and directional processes modulate automatic visual perspective-taking, and perspective-taking effects are strongly influenced by experimental context.

Visual perspective-taking involves an assessment of what or how another person sees a visual stimulus, independent of what or how we see that same stimulus ourselves. These processes are therefore central to Theory of Mind (ToM), and the ability to ascribe mental states (e.g., knowledge, beliefs, intentions, etc.) to the self and others. In recent years, researchers have become increasingly interested in the individual differences that predict an *observer’s* ability to take another person’s perspective. This busy field of research has identified numerous characteristics that modulate success on a variety of ToM tasks, including the observer’s age (e.g., Phillips et al., 2011), working memory, and inhibitory control skills (e.g., Bradford, Jentzsch, & Gomez, 2015; Brown-Schmidt, 2009; Cane, Ferguson, & Apperly, 2017; German & Hehman, 2006; Lin et al., 2010), attentional processes (Rubio-Fernández & Geurts, 2016), social skills (Brunyé et al., 2012; Ferguson et al., 2015; Kessler & Wang, 2012; Nielsen et al., 2015), mood (Converse et al., 2008), and cultural background (Wu & Keysar, 2007). In contrast, very little research has considered how characteristics of the *observed person* might influence perspective-taking success. The current study addresses this issue by examining how the age of an observed person (adult vs. child avatar) influences adults’ visual perspective-taking, particularly the degree to which they experience interference from their own (i.e., egocentric) or the other person’s (i.e., altercentric) perspective when responding from the “other” or “self” perspective, respectively.

A popular paradigm that has been used to examine visual perspective-taking is the “avatar” task, in which participants have to verify the number of discs in a visual scene according to either their own or a central on-screen avatar’s perspective. Crucially, in some trials the two perspectives are inconsistent (i.e., each sees a different number of discs), while in others they are consistent. Samson, Apperly, Braithwaite, Andrews, and Bodley Scott (2010) found that healthy adults can rapidly and accurately compute other people’s visual perspectives, or respond according to their own broader viewpoint (which may include objects that are hidden from the avatar’s view). Nevertheless, participants’ responses were slower and less accurate for trials in which judging what the avatar could see required them to inhibit their own visual perspective, and when judging what they could see required them to inhibit the avatar’s visual perspective. Thus, participants experienced difficulty ignoring the irrelevant perspective (i.e., either what they saw or what the avatar saw) when the two perspectives differed; performance on the task was influenced by both egocentric and altercentric tendencies.

While this pattern has been replicated numerous times (e.g., Catmur et al., 2016; Conway et al., 2017; Ferguson, Apperly, & Cane, 2017; Nielsen et al., 2015; Qureshi, Apperly, & Samson, 2010; Santiesteban et al., 2014), there has been much debate in the literature regarding whether the altercentric effect genuinely reflects interference from the avatar’s perspective (i.e., automatic mentalising), or whether it is driven by domain-general attentional cues based on directional features of the avatar (i.e., sub-mentalising; Heyes, 2014; Santiesteban et al., 2014). To test these alternatives, researchers have compared effects when the central avatar is replaced by a non-social (directional) cue (e.g., an arrow, lamp, or wall; Samson et al., 2010, Experiment 3; Nielsen et al., 2015; Santiesteban et al., 2014; Schurz et al., 2015) or when the avatar’s view of the stimulus is restricted (e.g., by opaque goggles/barrier, or an “invisibility” telescope; Furlanetto et al., 2016; Cole et al., 2016; Conway et al., 2017). Results are inconsistent across these studies, with some showing that altercentric interference is attenuated when a non-social (i.e., inanimate) agent is present or when they have a restricted view of the stimulus, therefore supporting a mentalizing account, but others revealing comparable inconsistency effects for inanimate and restricted view designs, thus supporting the dominant role of attentional processes.

The current study uses the avatar visual perspective-taking task to test whether the age of the observed person (adult vs. child avatar) influences adults’ visual perspective-taking performance. Therefore, while we do not directly aim to test mentalizing versus directional accounts of automatic perspective-taking, the results clearly have a bearing on this debate. Specifically, a purely attentional account would predict no difference between child and adult avatars since directional features (i.e., forehead, eyes, nose, etc.) are equated between avatars. In contrast, if we find that avatar age modulates altercentric interference this would suggest that participants have inferred different mental states for child and adult avatars, and therefore would support the role of mentalizing in this task.

Our age manipulation links to neuroimaging research that has revealed overlapping neural activation between self and other mentalizing when the person is considered to be similar to the self, but not when the person is different from the self (Davis et al., 1996; Mahajan & Wynn, 2012; Mitchell et al., 2006; Pfeifer et al., 2009). This pattern suggests that participants refer to their own perspective to understand how a similar person might be seeing, feeling, or thinking, and fits with a spontaneous perspective-taking mechanism that is especially pronounced when one feels socially connected to the other person (Smith & Mackie, 2016). In line with this, studies that have examined how similarity between the self and other influences mental state inferences report *greater* egocentric interference when people are taking the perspective of an ingroup member compared to an outgroup member (e.g., Simpson & Todd, 2017; Savitsky et al., 2011; Todd et al., 2011). In particular, Simpson and Todd (2017) adapted the avatar task described above by manipulating the group membership of the avatar, such that university affiliations and personality traits distinguished in-group from out-group members. Results revealed increased egocentric interference with in-group than out-group avatars, but no influence of avatar group membership on altercentric interference (though shared group membership did facilitate “other” perspective-taking on consistent trials). In the current study our choice to examine effects of the avatar’s age was based on research that has demonstrated an own-age bias, reflecting enhanced performance in a range of social perception tasks when the other person is in the same age category as the perceiver (e.g., Bailey et al., 2014; Melinder et al., 2010; Rhodes & Anastasi, 2012; Slessor et al., 2010; Slessor et al., 2014).

We complement the standard behavioral data collected in this paradigm by recording event-related brain potentials (ERPs) to examine the effects of perspective-taking and age of avatar in real-time. To date only one study has applied this technique to the avatar visual perspective-taking paradigm (McCleery et al., 2011); however, a growing number of studies have used ERPs to examine other aspects of ToM. Many of these studies have examined the brain’s response as participants answer explicit belief questions (e.g., “where does X think the Y is?”, e.g., Liu et al., 2004, 2009; Sabbagh & Taylor, 2000; Wang et al., 2008; Zhang et al., 2009), or passively observe pictorial sequences of events depicting beliefs and desires (e.g., Geangu et al., 2013; Kühn-Popp et al., 2013; Meinhardt et al., 2012), and have consistently demonstrated a positive-going late frontal slow wave (LFSW, ~300 ms onwards) when people are required to reason about others’ (false) beliefs versus reality. Though there is general agreement that differences on the LFSW reflect the key processes that distinguish mental states from reality (Liu et al., 2004; Sabbagh & Taylor, 2000), the exact mechanisms that underlie this component remain controversial due to the variety of paradigms and component definitions (i.e., time course or topography) that have been used in existing studies. Thus, deflections of the LFSW have been attributed to the experience of conflicting self/other perspectives (Jiang et al., 2016), the need to inhibit the self-perspective when inferring others’ beliefs (Zhang et al., 2009), and shifting between external stimuli and internal mental representations (Meinhardt et al., 2011).

More recently, researchers have reported effects of self-other processing on another ERP component, the P300, in a variety of social cognitive paradigms (see Knyazev, 2013 for a review). These studies typically manipulate the consistency of self-reference with auditory, visual, or sensory experiences (e.g., own name/face pairings, Cygan, Tacikowski, Ostaszewski, Chojnicka, & Nowicka, 2014; observed/intended actions, Deschrijver, Wiersema, & Brass, 2017; self/other touch, Deschrijver, Wieserma, & Brass, 2016). This work has consistently shown modulation of the P300 when processing self-relevant information, suggesting that this component indexes the distinction between self and other perspectives. However, in contrast to non-social oddball-type effects (e.g., Picton, 1992; Polich, 2007), these self-referenced effects reveal larger P300 amplitudes for self-*compatible* conditions compared to self-*incompatible* conditions. It has been suggested that this pattern reflects the increased need to resist interference when self and other perspectives are inconsistent, meaning that less resources are available to generate the P300 (Deschrijver et al., 2017). Thus, similar to the LFSW, self-referenced modulations of the P300 are likely to reflect both the social process of distinguishing self and other perspectives, and the recruitment of higher-order cognitive processes to evaluate self-related stimuli, and support increased allocation of attention and conflict resolution (Conde et al., 2015; Tacikowski & Nowicka, 2010). We note that the existing literature does not provide a clear distinction between the social and cognitive contributions to LFSW and P300 components, and indeed some researchers have reflected on whether the two components might reflect common processes given the overlapping time windows and scalp distributions (Jiang, Li, Li, Wang, Cao, & Li, 2016).

One study has directly explored the neural basis of visual perspective-taking by recording ERPs and estimating the neural sources while participants completed an auditory-visual version of the avatar visual perspective-taking task (McCleery et al., 2011; e.g., “she sees N” - [image]). Results revealed that perspective and consistency modulated numerous ERP components, including the amplitude of the P200 (larger amplitude over occipital midline electrodes for self-inconsistent trials than any other trial types), and the latency and amplitude of a middle latency component (referred to as TP450; longer peak latencies for other- than self-perspective trials, particularly other-inconsistent, and larger peak amplitudes for consistent compared to inconsistent trials). Consistency also modulated the LFSW between 600–800 ms (consistent > inconsistent). The authors suggest that modulations of the P200 component reflect strategic allocation of visual attention, since the self-inconsistent condition is the only trial type that requires attention to be divided between both walls (i.e., in front and behind the avatar). Crucially, the latency of deflections of the TP450 were attributed to the processing costs of calculating the avatar’s perspective (which are highest in the other-inconsistent condition), with source analyses linking TP450 effects to the temporal parietal cortex (Saxe & Kanwisher, 2003). These TP450 effects, showing influences of perspective and consistency, are therefore compatible with the interference effects seen on the P300 in the self-other tasks described above. Finally, the consistency effect on the LFSW amplitude is interpreted as reflecting the recruitment of executive functions to manage conflicting perspectives (localized to the right frontal cortex), and is therefore consistent with the ERP studies of belief processing, described above (e.g., Jiang et al., 2016; Zhang et al., 2009).

In this paper, we present three experiments that systematically examine whether and how age of avatar influences adults’ visual perspective-taking. In our first experiment, we recorded ERPs and behavioral responses while participants completed a version of the avatar visual perspective-taking task, with age of avatar manipulated between two groups (adult vs. child). In line with previous studies we predicted that this task would elicit both egocentric and altercentric interference effects, reflected in reduced accuracy and increased reaction times when the two perspectives were in conflict (i.e., a main effect of consistency). Replicating previous work, we also expected this consistency effect on reaction times to be larger when cued to take the avatar’s perspective than when cued to take the self perspective (i.e., a perspective × consistency interaction), reflecting the heightened need to inhibit irrelevant perspectives, with greater interference from the egocentric perspective than the altercentric perspective. Given the converging effects seen across previous ERP investigations of ToM processing, our ERP analyses focused on three key components: P200 (associated with perceptual processing), P300, and LFSW (reflecting self-other distinctions and the management of self-other conflicts). Thus, if deflections of the P200 reflect relatively low-level strategic allocation of visual attention, we expected to replicate McCleery et al.’s pattern of maximal amplitude for self-inconsistent trials (due to divided attention on these trials). More importantly, we expected the higher-level processes of distinguishing self/other perspectives and inhibiting the alternative perspective to be reflected in reduced P300 and LFSW amplitudes for inconsistent trials, with a larger consistency effect on these components for other- than self-perspective trials since the self (egocentric) perspective causes greater interference (and thus less available cognitive resources) than altercentric intrusions (as in Deschrijver et al., 2017). McCleery et al. used source localization to make a clear distinction between the mechanisms underlying their mid-latency TP450 component (representing social self-other conflict processes) and the LFSW waveform (executive processes to manage conflict). However, due to paradigm and component differences in the current studies, and based on the existing literature that implicates both ToM *and* executive processes, we do not tie our predictions on the P300 and LFSW components to distinct social/cognitive mechanisms.

Crucially, if the age of avatar manipulation activates distinct mental state processing mechanisms for similar and dissimilar others then we would expect to see modulations of these perspective-taking effects according to the age of avatar, via an own-age bias (i.e., an avatar × consistency interaction, or an avatar ×perspective × consistency interaction). Such modulations should be limited to response times, P300 amplitude, and LFSW amplitude since these measures have been shown to directly reflect high-level self-other processing and conflict (note that we do not expect age of avatar to influence low-level attention allocation, as measured by P200 amplitude). We expected this effect to reflect reduced processing of the other perspective when a child (i.e., dissimilar) versus adult (i.e., similar) avatar was present, and for it to be manifest in a reduced or absent altercentric interference effect (similar to previous studies that have manipulated avatar animacy/view, e.g., Furlanetto et al., 2016; Schurz et al., 2015), and/or a larger egocentric interference effect (similar to the ingroup effect seen in Simpson & Todd, 2017). In contrast, a purely directional account that does not activate spontaneous mentalizing would not predict any differences in processing between adult and child avatars, since directional features are matched between avatars.

## Experiment 1

### Method

#### Participants

A total of 38 English-speaking Caucasian students from the University of Kent took part in the study. Four of these participants were excluded due to poor accuracy on the task (<50%) or poor quality of EEG data (resulting in a trial loss of > 40%). Thus, the final sample included 34 participants (24 female; 29 right-handed; M_age_ = 20.5 years), split equally between the adult and child avatar groups, and matched between groups on gender and age. This sample size was determined *a priori* to match the sample size used in McCleery et al.’s (2011) ERP avatar visual perspective-taking task (N = 17) in each of our avatar age groups.

All participants completed the Empathy Quotient (Baron-Cohen & Wheelwright, 2004), a 40-item self-report questionnaire that assesses empathy and social aptitude. In addition, all participants completed the Simon task (Simon & Wolf, 1963), consisting of 80 trials (40 consistent/40 inconsistent), and an inhibitory control score was calculated by subtracting reaction times for correct responses on consistent trials from inconsistent trials. Age of avatar groups was therefore statistically matched on participants’ gender (12 females and five males in each group), age (adult *M* = 21.5; child *M* = 19.5; *t* = 1.57), empathy quotient score (adult *M* = 40.8; child *M* = 37.9; *t* = .88), and inhibitory control score (adult *M* = 21.4; child *M* = 19.0; *t* = .88).

#### Materials

**Fig. 1:**
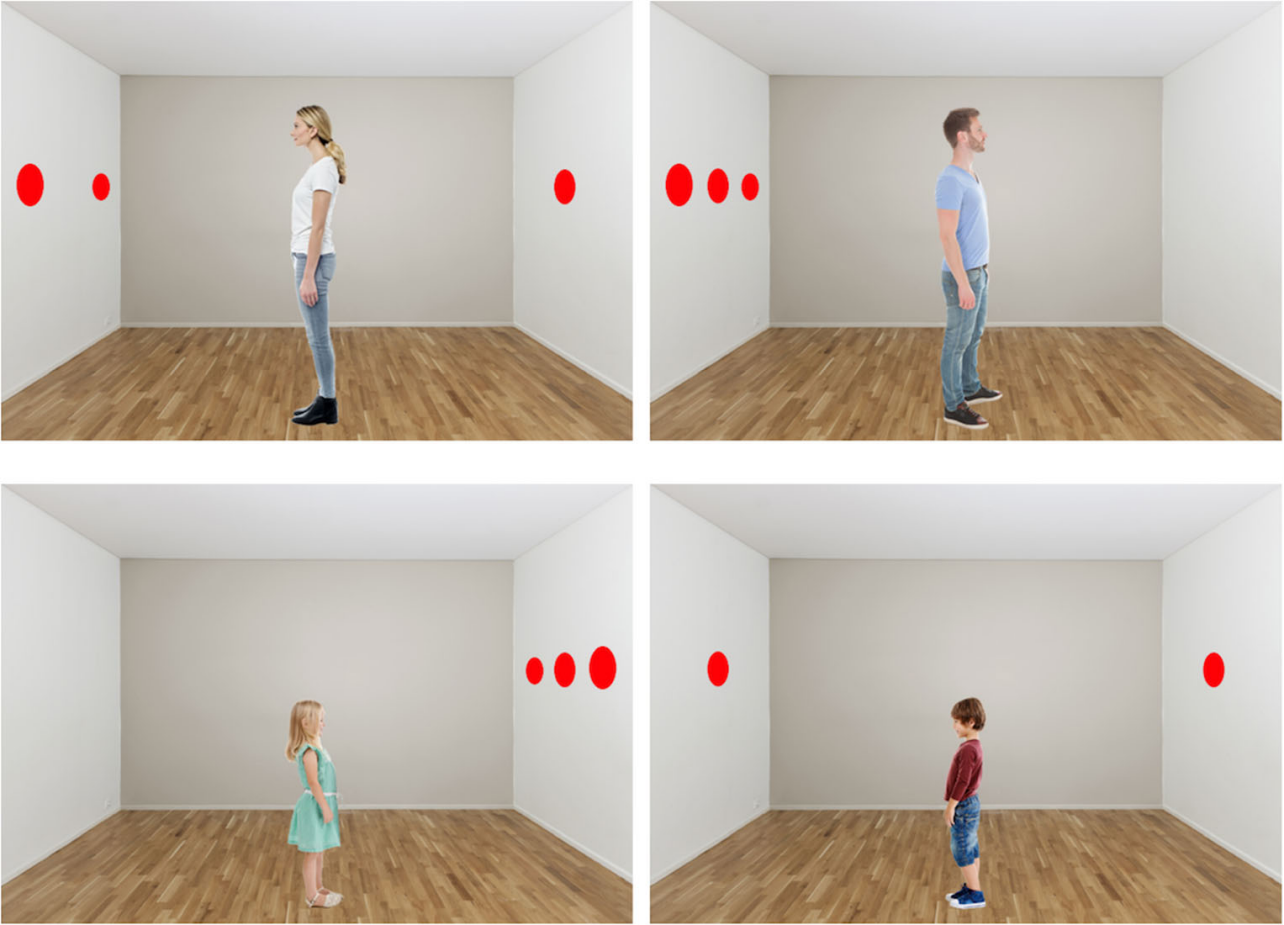
Examples of the visual stimuli, showing the age of avatar manipulation, and different configurations of discs on the walls. Note that the avatar’s gender always matched the participant’s gender

Participants took part in a visual perspective-taking task (adapted from Samson et al., 2010) while EEG activity was continuously recorded. The visual stimuli included a 3D lateral view of a room, where the ceiling, floor, left, right and back walls were visible. Red discs were displayed on one or two of the left/right walls. The number and position of discs changed on each trial. In addition, a realistic human avatar was standing in the center of the room, facing either the left or right wall. The avatar’s gender always matched the participant’s gender, but half the participants saw an adult-like avatar, and the other half saw a child-like avatar.^1^ On half the trials, the avatar’s orientation meant that s/he saw the same number of discs as the participant (consistent condition), and on the other half, the avatar’s orientation meant that s/he could not see some of the discs that were visible to the participant (since they were placed on the wall behind the avatar; inconsistent condition). See Fig. 1 for examples of these visual stimuli, and the Open Science Framework for the full set of materials (https://osf.io/bqw4h).

To ensure that the directional features were matched between child and adult avatars, the stimuli were pre-tested using a Posner paradigm (Posner, 1980). Sixteen participants (M_age_ = 24.8 years) completed a total of 96 trials in a within-subjects design that crossed avatar (child vs. adult) and gaze-cue validity (valid vs. invalid), thus 24 trials in each condition. Trials began with a central fixation cross in the empty 3D room (700 ms), followed by the central avatar facing left or right (i.e., the gaze cue; 300 ms), and finally a single red disc appeared on the left or right wall (replicating the position of discs used in the main task) until a response was made. Correct response times were analyzed using a within-subjects 2 × 2 ANOVA, crossing avatar (adult vs. child) and gaze-cue validity (valid vs. invalid). Results revealed a significant main effect of gaze-cue validity (valid = 311 ms vs. invalid = 336 ms, *F*(1, 15) = 141.3, *p* < .001, _p_η^2^ = .9), but no main effect of avatar (*F* = .01, *p* = .91), or an interaction between the two variables (*F* = .51, *p* = .49).

#### Procedure

Participants were informed about the EEG procedure and experimental task. Their task was to verify the number of discs that were visible either according to their own perspective (self-perspective condition), or according to the avatar’s perspective (other-perspective condition). Trials were either matching or mismatching. On matching trials, the cue digit corresponded to the number of discs that could be seen from the cue perspective for the target image. On mismatching trials, the cue digit did not correctly correspond to the number of discs that could be seen from the cue perspective. After electrode application they were seated in a booth where they read the materials from a computer screen. The experiment was controlled using E-Prime software.

**Fig. 2:**
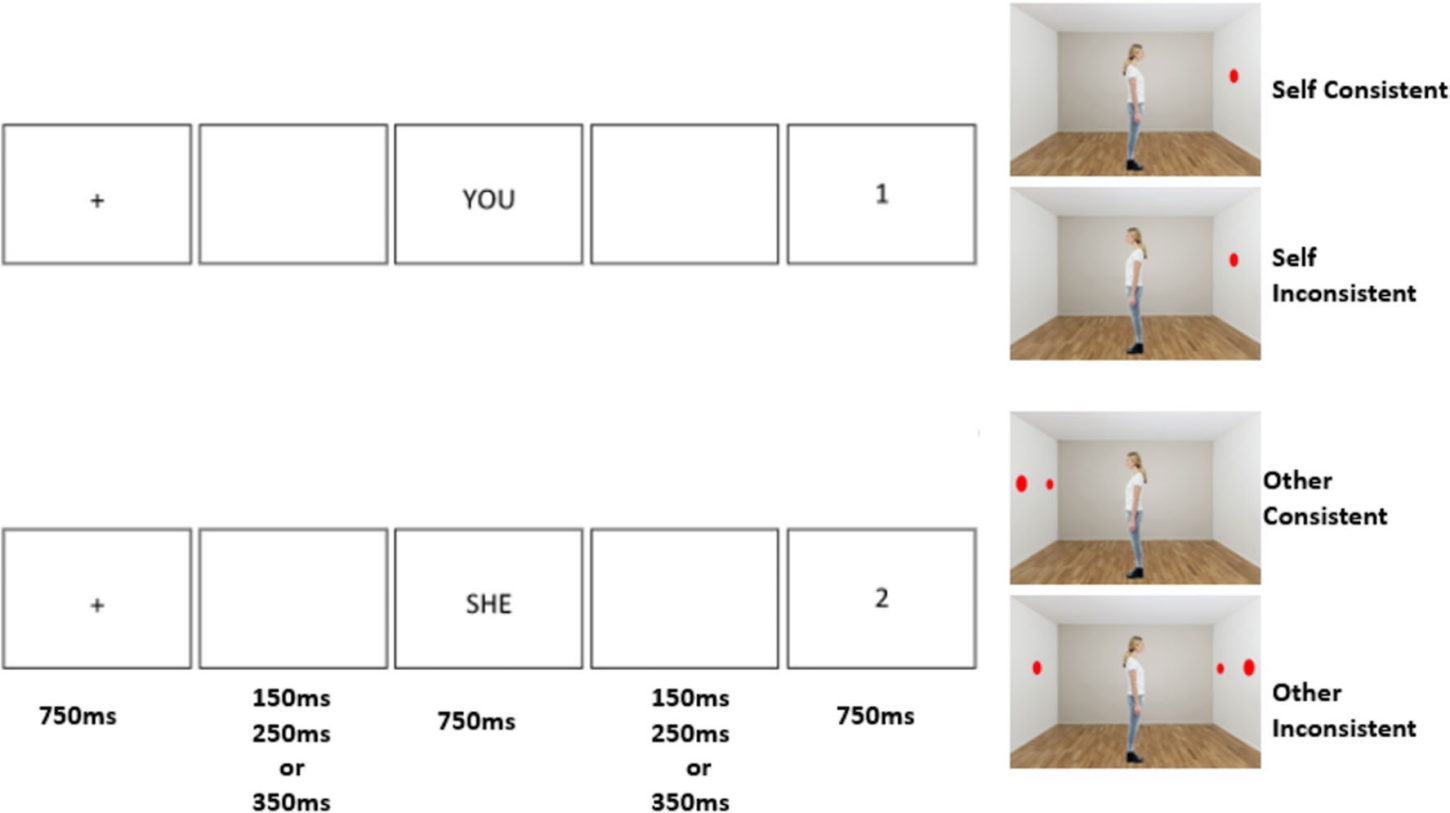
Schematic trial sequence of visual displays presented to participants in the visual perspective-taking task

Each trial began with a fixation cross in the center of the screen for 750 ms. Following a blank screen inter-stimulus interval (ISI) of 150 ms, 250 ms, or 350 ms,^2^ the word “YOU” or “SHE/ HE” was presented for 750 ms. This informed participants whether to respond to the current trial according to their own or the avatar’s perspective. Following a second blank screen ISI, a digit between 0 and 3 was shown in the center of the screen for 750 ms. This indicated the number of discs the participant needed to verify, according to the given perspective. Finally, the target image of the room, avatar, and discs (650 × 480 pixels) appeared centrally on-screen. Participants were instructed to judge whether the number of discs in the target image matched the preceding digit according to the cued perspective or not, using keys “z” and “m” (key associations were counterbalanced across participants). Participants were asked to respond as quickly and accurately as possible. The screen advanced to the next trial once a keyboard response had been detected or for a maximum of 2000 ms (see Fig. 2).

Participants completed a practice block of 26 trials, followed by the main task, which consisted of 12 blocks, each with 52 trials. In total there were 288 matching trials, 288 mismatching trials, and 48 “filler” trials (where no discs were displayed on either wall so that the disc number 0 was sometimes correct for self-perspective trials). Participants were asked to respond according to their own perspective on half the trials, and to respond according to the avatar’s perspective on the other half. Of these, half were consistent trials, where the avatar and participant saw the same amount of discs on the wall, and half were inconsistent trials, where the avatar and participants’ views were different. Trials were presented in a pseudorandom order mixing self and other perspectives, such that no more than four consecutive trials that tapped the same perspective, and no more than three consecutive trials tapped the same perspective-consistency condition. No complete stimulus repetitions (i.e., same perspective cue and image) were included. The full experiment lasted for about 80 min.

In sum, three independent variables were manipulated in a 2 (Consistency: consistent vs. inconsistent) × 2 (Perspective: self vs. other) × 2 (Avatar: adult vs. child) mixed design, with Consistency and Perspective being within-subjects and Avatar being between-subjects. Effects were analyzed at the target image, on accuracy of responses, response time, and the ERP components as detailed below.

#### Electrophysiological measures

EEG activity was recorded continuously using a Brain Vision Quickamp amplifier system with a 62-channel ActiCap, over midline electrodes Fz, Cz, CPz, Pz, POz, and Oz, over the left hemisphere from electrodes Fp1, AF3, AF7, F1, F3, F5, F7, FC1, FC3, FC5, FC7, C1 C3, C5, T7, CP1, CP3, CP5, TP7, A1, P1, P3, P5, P7, PO3, PO7, PO9, O1, and from the homologue electrodes over the right hemisphere. EEG data were referenced online to electrode FCz, and grounded to electrode AFz. EEG and EOG recordings were sampled at a rate of 500 Hz. Electrode impedances were kept at <25 KΩ.

**Fig. 3:**
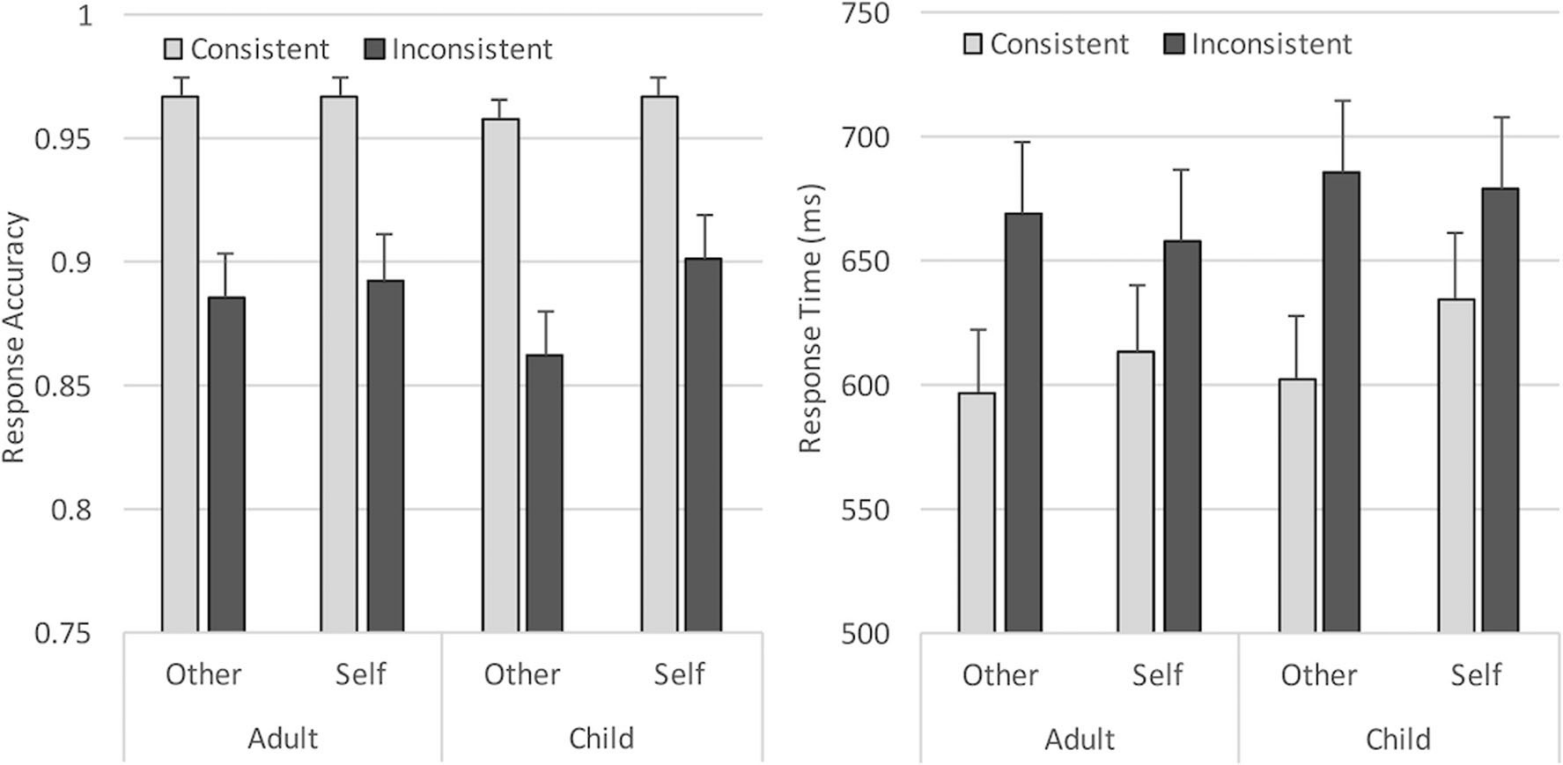
Mean response accuracy and response times for each condition in Experiment 1. Error bars show standard errors

Brain Vision Analyzer 2 software was used to prepare the data prior to analysis. First, noisy or faulty electrodes were interpolated from surrounding channels (a maximum of three channels), then all channels were re-referenced offline to an average reference (excluding eye channels and mastoids) and the EEG signal was band-pass filtered (0.3–40 Hz, 12 dB/oct). Data containing blinks and horizontal eye movements were corrected using semi-automatic ocular Independent Components Analysis (ICA) correction (which removed an average of three components per participant), then the data was segmented into epochs of 1,100 ms time-locked to picture onset (-100 – 1,000 ms). Any trial where the participant made an incorrect picture judgment was eliminated from further ERP analysis, then each trial was individually inspected to identify and discard trials with non-ocular artifacts (drifts, channel blockings, EEG activity exceeding ± 75μV), using a semi-automatic artifact rejection algorithm. Together, these procedures resulted in an average trial loss of 11.3% per participant, and an average of 64 accepted segments per condition/participant. A 2 (Perspective) × 2 (Consistency) × 2 (Avatar) ANOVA testing trial loss across conditions revealed no difference between avatar conditions (*p* = .71) or perspective (*p* = .15) or any interactions (all *p*s > .1), but significantly less accepted segments per participant for inconsistent trials than consistent trials (61 vs. 67; *F*(1, 32) = 65.23, *p* < .001, _p_η^2^ = .67), due to differences in accuracy in these conditions (see behavioral results below). Finally, the signal at each electrode site was aligned to a 100-ms baseline, then averaged separately for each experimental condition (Fig. 3).

#### ERP data analysis

**Fig. 4:**
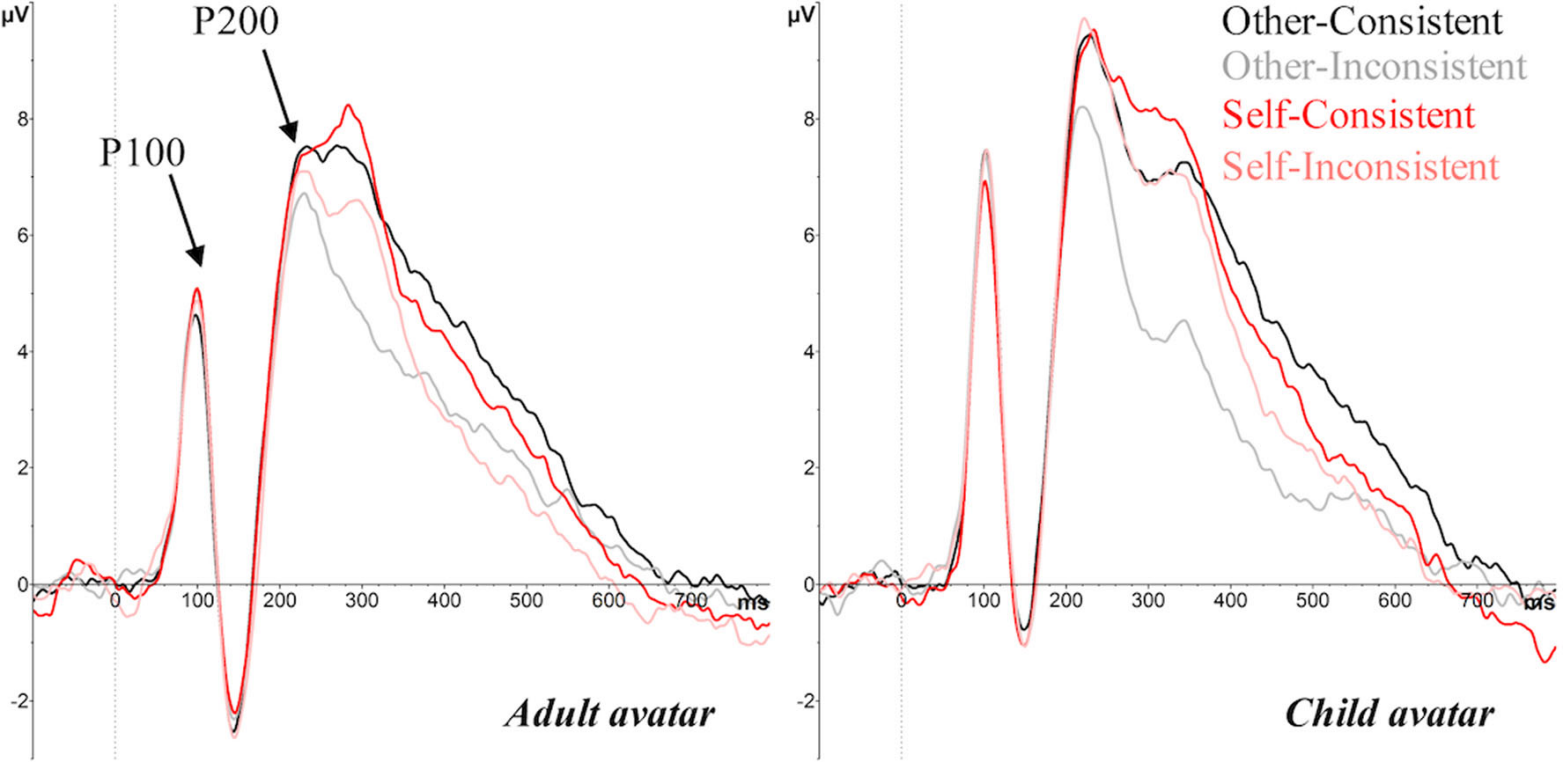
Grand average ERPs over the central occipital lobe elicited by the target image for other consistent, other-inconsistent, self-consistent and self-inconsistent conditions, showing the P100 and P200 for the adult avatar (left panel) and the child avatar (right panel), in Experiment 1

**Fig. 5:**
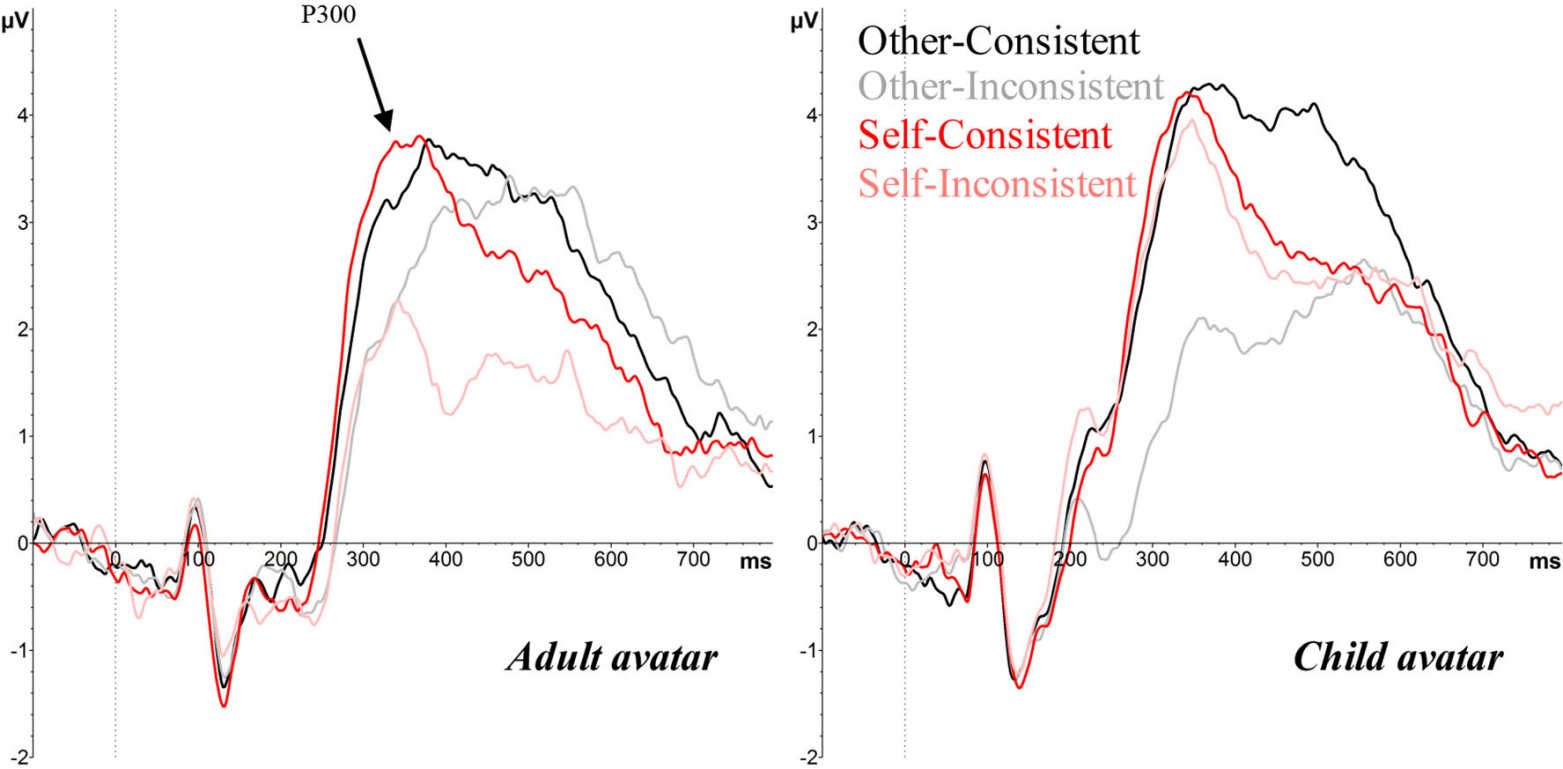
Grand average ERPs over the central parietal lobe elicited by the target image for other-consistent, other-inconsistent, self-consistent and self-inconsistent conditions, showing the P300 for the adult avatar (left panel) and the child avatar (right panel), in Experiment 1

**Fig. 6:**
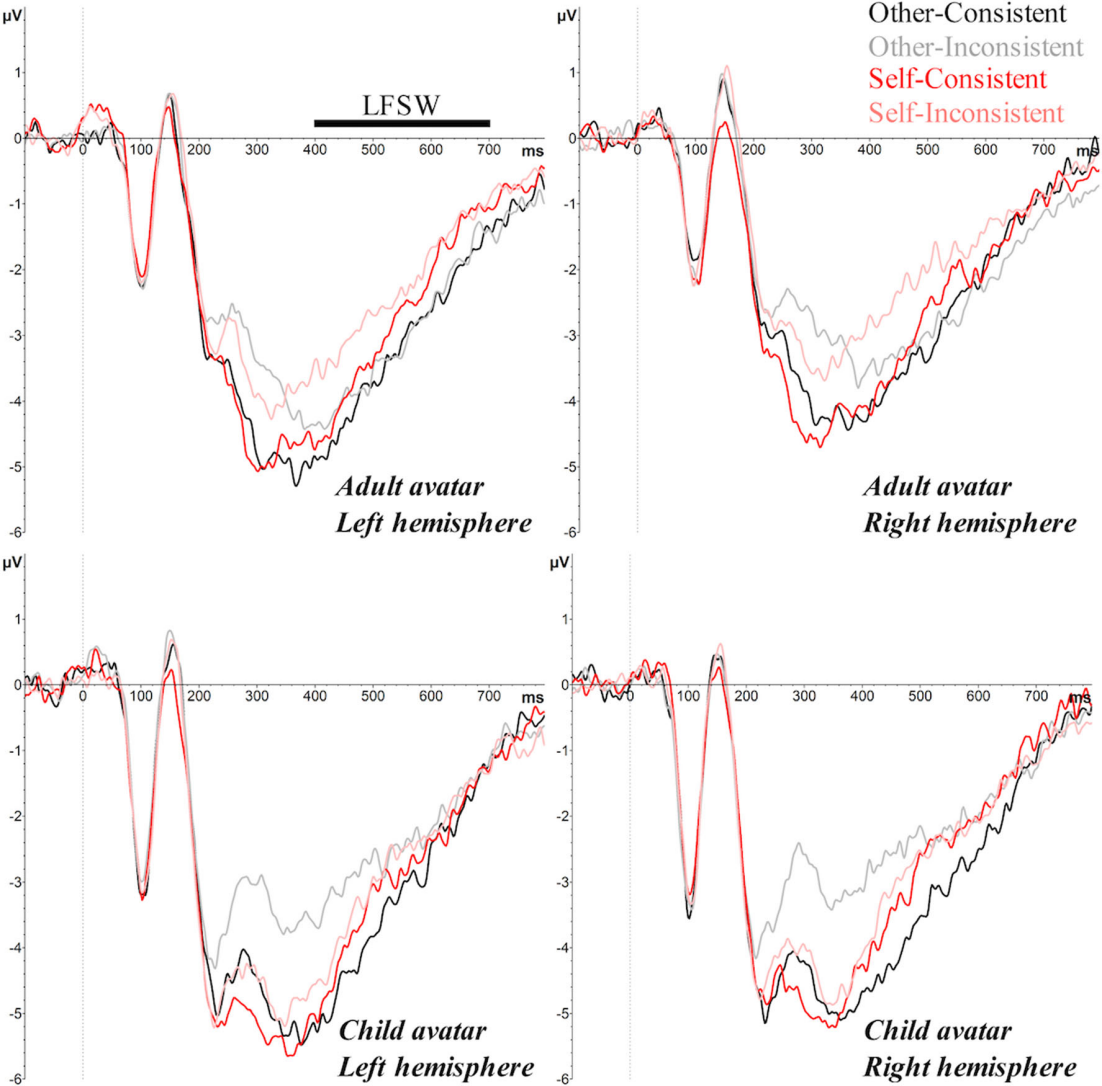
Grand average ERPs over the left (left panels) and right (right panels) frontal lobes elicited by the target image for other-consistent, other-inconsistent, self-consistent and self-inconsistent conditions, showing the LFSW for the adult avatar (top panel) and the child avatar (bottom panel), in Experiment 1

**Fig. 7:**
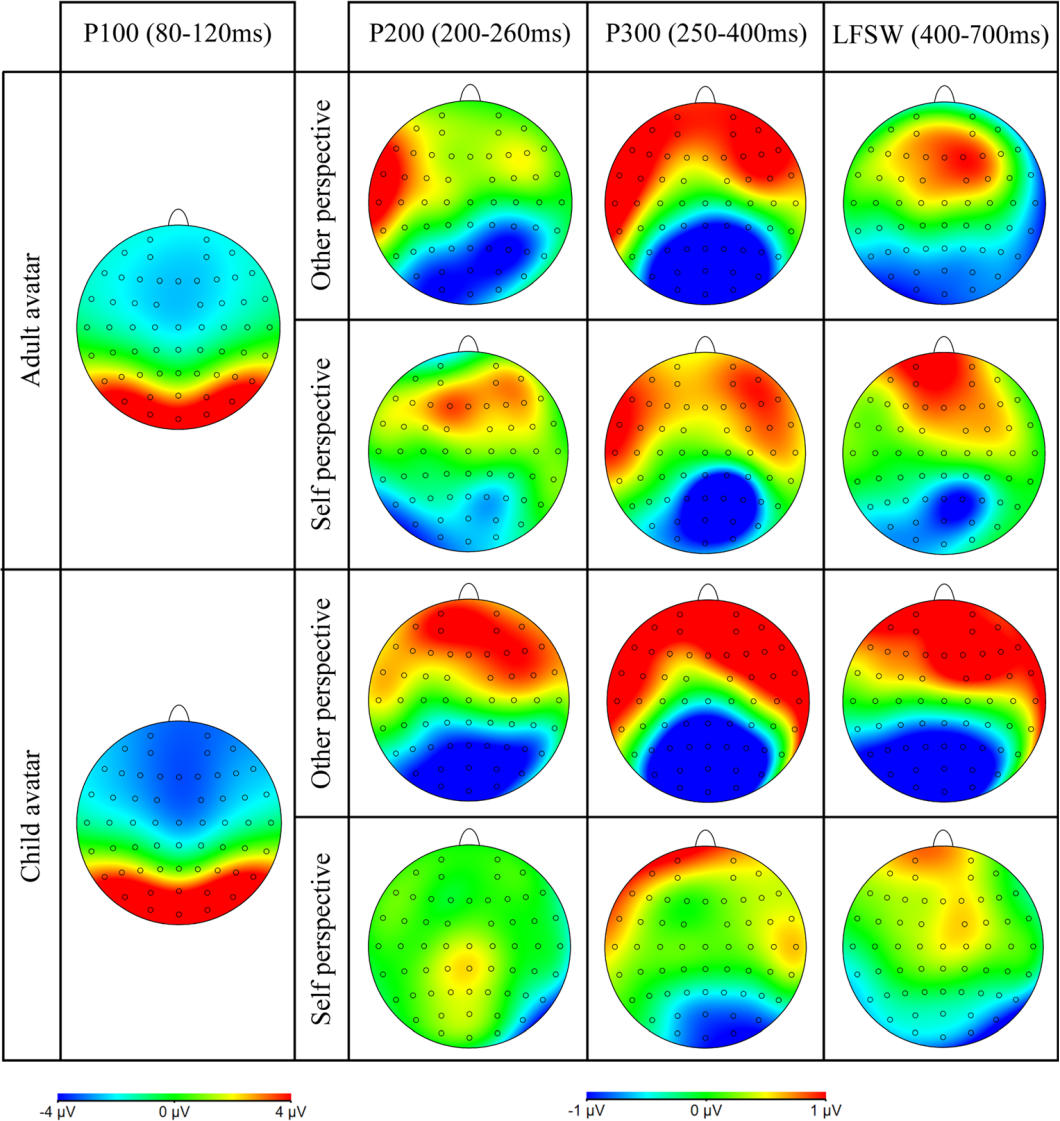
Topographic maps show the ERP waveform for each component of interest. Data for the P100 shows the age of avatar effect, averaged over condition. Data for the P200, P300 and LFSW show the consistency effect (i.e. inconsistent minus consistent), separately for each avatar and perspective condition

Three ERP components were identified for analysis, based on previous research that has examined perspective and consistency effects in a visual perspective-taking task (McCleery et al., 2011), and ERP studies of self-referential processing (e.g., Cygan et al., 2014; Deschrijver et al., 2016, 2017). Thus, our analyses focused on the peak amplitude of the P200 (a positive-going component, peaking between 200–260 ms over central occipital electrode sites, associated with perceptual processing, see Fig. 4), the peak latency and amplitude of the P300 (a positive-going component peaking between 250–400 ms over central parietal electrode sites, reflecting self-other distinctions, see Fig. 5), and the mean amplitude over a late frontal slow-wave (LFSW, between 400–700 ms over the left and right lateral frontal cortex, reflecting management of self-other conflicts, see Fig. 6). We note that our P300 component is consistent with research in the field of self-referential processing and is comparable to the TP450 component seen in McCleery et al., and attribute the slightly different topography and peak latency to the fact that stimuli in McCleery et al. were presented in a multi-modal auditory-visual format (e.g., “she sees N” - [image]), whereas all stimuli in the current study were presented in a visual sequence (as is typical in this paradigm, e.g., Samson et al., 2010; Santiesteban et al., 2014). In addition, we conducted exploratory analyses on the P100 amplitude (an early positive-going component peaking between 80–120 ms over central occipital electrode sites), since visual inspection of the ERP waveforms suggested a group difference on this component (see Figs. 4 and 7). The P100 is a sensory response to visual stimuli, and is sensitive to stimulus parameters, such as size and luminance, thus we tested for between-groups differences here to quantify early differences in the waveform due to physical differences between adult and child avatar stimuli, which may contaminate subsequent ERP effects.

The electrodes used to measure each component were as follows: left frontal: AF7, F7, F5; right frontal: AF8, F6, F8; central parietal: CP1, CP2, CPz, Pz, P1, P2; central occipital: POz, PO3, PO4, Oz, O1, O2. Peak amplitudes (P100, P200, and P300), latencies to peak amplitudes (P300), and mean amplitudes (LFSW) were identified using the time intervals defined above using Brain Vision Analyzer’s automatic peak detection algorithm and measured for each individual electrode in the relevant conglomerate, then averaged within the relevant region for each participant and condition. For the statistical analysis of amplitude (and latency) data over central occipital and central parietal components (P100, P200, and P300), ANOVAs with variables Perspective (self vs. other), Consistency (consistent vs. inconsistent), and Avatar (adult vs. child) were conducted. The LFSW was analyzed as the mean amplitude over lateral frontal sites using an ANOVA with variables Hemisphere (left vs. right), Perspective (self vs. other), Consistency (consistent vs. inconsistent), and Avatar (adult vs. child).

### Results

Accuracy and response times for matching trials were analyzed using separate 2 × 2 × 2 analyses of variance (ANOVA), with Perspective (Self vs. Other) and Consistency (Consistent vs. Inconsistent) as within-subjects variables, and Avatar (Adult vs. Child) as the between-subjects variable. Note that due to space constraints, only significant or marginal (*p* <= .06) effects are presented in the text throughout this manuscript. Full statistical effects for each experiment and measure are summarised in the Appendix, and full data for each experiment and measure are available on the Open Science Framework (https://osf.io/bqw4h/?view_only=e275ad0e97dc42b7b6dcf17e089df06d). In line with standard procedures, behavioral analyses did not exclude trials based on ERP preprocessing. Incorrect picture verification responses and trials where the participant did not respond to the image in the given 2,000 ms were excluded from the response-time analysis (5.5%), which was measured from the onset of the picture. Resulting mean response accuracy and response times for each condition are shown in Fig. 3.

#### Response accuracy

The ANOVA revealed a significant main effect of Perspective (*F*(1, 32) = 4.20, *p* = .049, _p_η^2^ = .12), reflecting higher accuracy when participants responded according to their own (*M* = 93.2%) compared to the avatar’s perspective (*M* = 91.8%). In addition, a significant main effect of Consistency (*F*(1, 32) = 72.19, *p* < .001, _p_η^2^ = .69) showed that accuracy was higher when participants shared the same visual perspective with the avatar (*M* = 96.5%), compared to when the two perspectives were inconsistent (*M* = 88.5%). Neither Avatar or the interactions were significant (all *F*s < 2.25, *p* > .11).

#### Response times

The ANOVA showed a significant main effect of Consistency (*F*(1, 32) = 93.62, *p* < .001, _p_η^2^ = .75), with responses being slower when perspectives were inconsistent (*M* = 673 ms) compared to when perspectives were consistent (*M* = 612ms). In addition, Perspective interacted significantly with Consistency (*F*(1, 32) = 16.65, *p* < .001, _p_η^2^ = .34). Bonferroni corrected *post hoc* tests revealed that the Consistency effect was larger when taking the Other perspective (*t*(33) = 11.56, *p* < .001; inconsistent *minus* consistent = 77 ms), compared to when taking the Self perspective (*t*(33) = 5.47, *p* < .001; inconsistent *minus* consistent = 44 ms). These results replicate previous studies and show that participants experienced both egocentric and altercentric interference, though intrusions from one’s own knowledge were significantly larger (paired-samples t-test comparing consistency effect in each perspective condition: *t*(33) = 4.04, *p* < .001). Neither the main effect of Perspective, Avatar, or the other interactions were significant (all *F*s < 1.72, *p*s > .2).

#### ERP effects

##### P100

The analysis of P100 amplitude revealed a significant effect of Avatar (*F*(1, 32) = 4.73, *p* = .037, _p_η^2^ = .13), with the child avatar eliciting a larger amplitude (*M* = 7.57 μV) than the adult avatar (*M* = 5.21 μV). Since the P100 is known to reflect low-level perceptual analysis, we attribute this avatar effect to physical differences between the child and adult stimuli (e.g., luminance, spatial frequency; Linkenkaer-Hansen et al., 1998). There were no other significant main effects or interactions (all *F*s < 2.82, *p*s > .1).

##### P200

The ANOVA on P200 amplitude revealed a significant main effect of Perspective (*F*(1, 32) = 9.99, *p* < .003, _p_η^2^ = .24), reflecting a larger P200 amplitude on self (*M* = 9.36 μV) than other trials (*M* = 8.85 μV), and a significant main effect of Consistency (*F*(1, 32) = 27.66, *p* < .001, _p_η^2^ = .46), reflecting a larger P200 amplitude on consistent (*M* = 9.47 μV) compared to inconsistent trials (*M* = 8.75 μV). In addition, a significant interaction between Perspective and Consistency (*F*(1, 32) = 7.83, *p* < .01, _p_η^2^ = .2) was found. Bonferroni corrected *post hoc* tests revealed that the Consistency effect was only significant when participants were cued to take the other perspective (*t*(33) = 5.66, *p* < .001), and not when cued to take their own perspective (*t*(33) = 1.71, *p* = .098). This pattern suggests a robust egocentric interference effect, but a weaker or absent altercentric interference effect. The effect of Avatar and the remaining interactions were not significant, all *F*s < 3.07, *p*s > .09.

##### P300

The ANOVA on latencies revealed a significant main effect of Perspective (*F*(1, 32) = 25.26, *p* < .001, _p_η^2^ = .44), with longer peak latencies in other (*M* = 355 ms) than self (*M* = 341 ms) trials. There was no significant main effect of Consistency or Avatar, or any interactions (all *F*s < 2.57, *p* > .119).

Analysis of P300 amplitude revealed a significant main effect of Consistency (*F*(1, 32) = 104.63, *p* < .001, _p_η^2^ = .77), with consistent trials (*M* = 4.89 μV) eliciting a larger amplitude than inconsistent (*M* = 3.65 μV). Interestingly, a significant interaction between Perspective and Avatar (*F*(1, 32) = 4.80, *p* = .036, _p_η^2^ = .13) was found, subsumed under a significant three-way interaction between Perspective, Consistency, and Avatar (*F*(1, 32) = 5.41, *p* = .026, _p_η^2^ = .15). Follow-up analyses examined effects for adult and child avatars separately. The adult avatar condition showed only a significant consistency effect (*F*(1, 16) = 39.50, *p* < .001, _p_η^2^ = .71), with consistent trials (*M* = 4.68 μV) eliciting a larger P300 amplitude than inconsistent trials (*M* = 3.50 μV). In contrast, the child avatar condition revealed significant main effects of Perspective (*F*(1, 16) = 21.66, *p* < .001, _p_η^2^ = .58; self *M* = 4.87 μV vs. other *M* = 4.04 μV), and Consistency (*F*(1, 16) = 72.10, *p* < .001, _p_η^2^ = .82; consistent *M* = 5.10 μV vs. inconsistent *M* = 3.81 μV). Moreover, the Perspective × Consistency interaction was significant (*F*(1, 16) = 19.43, *p* < .001, _p_η^2^ = .55). Bonferroni corrected *post hoc* tests revealed a significant consistency effect when participants were taking the other perspective (*t*(16) = 7.73, *p* < .001; consistent = 5.14 μV vs. inconsistent = 2.93 μV), but not when taking the self perspective (*t*(16) = 1.57, *p* = .136; consistent = 5.04 μV vs. inconsistent = 4.69 μV).

##### LFSW

The ANOVA revealed a significant main effect of Hemisphere (*F*(1, 32) = 5.02, *p* = .03, _p_η^2^ = .14), with a larger, more negative-going amplitude over the left hemisphere (*M* = -2.80 μV) than the right hemisphere (*M* = -2.38 μV). There was also a significant main effect of Consistency (*F*(1, 32) = 8.90, *p* = .005, _p_η^2^ = .22; consistent < inconsistent), and a main effect of Perspective (*F*(1, 32) = 6.28, *p* = .02, _p_η^2^ = .16; other < self). Similar to the P300 component, the three-way interaction between Perspective, Consistency, and Avatar was significant (*F*(1, 32) = 7.45, *p* = .01, _p_η^2^ = .19). Further analyses examined effects for adult and child avatars separately, and showed that the Perspective × Consistency interaction was only significant in the child avatar condition (*F*(1, 16) = 5.15, *p* < .05, _p_η^2^ = .24), and not in the adult avatar condition (*F*(1, 16) = 2.43, *p* = .14). Bonferroni corrected *post hoc* tests in the child avatar group revealed a significant consistency effect when participants were cued to take the avatar’s perspective (*t*(16) = 2.81, *p* = .01; consistent = -3.12 μV vs. inconsistent = -2.26 μV), but not when they were cued to use the self perspective (*t*(16) = .77, *p* = .45; consistent = -2.68 μV vs. inconsistent = -2.53 μV).

To further investigate whether the condition effects observed on the P300 and LFSW components can be differentiated, we ran an exploratory ANOVA that crossed Component (P300 vs. LFSW) × Site (Anterior vs. Posterior^3^) × Perspective (Self vs. Other) × Consistency (Consistent vs. Inconsistent) × Avatar (Adult vs*.* Child). This analysis showed a significant interaction between Component and Site (*F*(1, 32) = 26.63, *p* < .001, _p_η^2^ = .45), reflecting a significantly larger positivity over posterior sites for the P300 compared to the LFSW component. More importantly, this effect was subsumed under three-way interactions that revealed statistically different topographic distributions of condition effect between the two components. A significant Component × Site × Consistency interaction (*F*(1, 32) = 43.42, *p* < .001, _p_η^2^ = .58) showed that the consistency effect was significantly larger on the P300 component than the LFSW component over posterior (*t*(33) = 11.74, *p* < .001) and anterior sites (*t*(33) = 4.93, *p* < .001), though this difference was greater over posterior sites. Additionally, a significant Component × Site × Perspective interaction (*F*(1, 32) = 48.67, *p* < .001, _p_η^2^ = .60) revealed different effects of Perspective between P300 and LFSW components over posterior (*t*(33) = 6.63, *p* < .001) and anterior sites (*t*(33) = -4.93, *p* < .001). These findings provide some tentative evidence to suggest that the two components, emerging in consecutive but non-overlapping time windows, may reflect distinct stages of processing.

In summary, Experiment 1 replicated previous research in showing that both egocentric and altercentric biases interfered with visual perspective-taking, though the altercentric effect was smaller than the egocentric effect. Crucially, our ERP data revealed the first evidence that age of avatar modulates these effects; effects consistent with egocentric and altercentric intrusions were evident on P300 and LFSW amplitudes for adult avatars (i.e., increased amplitudes on consistent vs. inconsistent trials for both other and self perspectives), but altercentric effects on these components were attenuated with a child avatar (i.e., increased amplitudes on consistent vs. inconsistent trials only for the other perspective). These findings provide initial evidence that participants inferred different mental states for child and adult avatars, possibly due to an own-age bias, which facilitated spontaneous perspective-taking for a similar age other, but weakened perspective-taking for a dissimilar age other.

Nevertheless, age of avatar did not modulate behavioural responses, as the hypothesized Perspective × Consistency × Avatar interaction was not significant on the reaction time measure. When reflecting on why such effects did not emerge on behavioural measures it is important to note that these results were revealed when age of avatar was manipulated between groups, when participants were tested on a high number of trials, and when they were instructed to respond according to both self *and* other perspectives. Although we reduced influences from individual differences on participants’ responses by matching the adult and child avatar groups across numerous key measures (i.e., gender, age, empathy, inhibitory control), it is possible that other unexpected differences existed between the two groups. In addition, by testing both self and other perspectives within the same experiment, the difference between self and avatar perspectives was made salient, and computing the avatar’s perspective on a given trial was task-relevant. This design makes it difficult to conclude that modulations of altercentric interference (i.e., on the self trials) reflect genuine influences on *automatic* perspective-taking, and thus might reflect simple carry-over effects from having to compute the avatar’s perspective on ‘other’ perspective trials. Indeed, whether participants were asked to verify the number of discs according to both their own *and* the avatar’s perspective, or whether judgments were limited to their own perspective *only*, has been identified as a key methodological difference between previous studies that do or do not show mentalising effects (see Cole et al., 2016; Conway et al., 2017), since automaticity can only be certain when the other perspective is task-irrelevant. This observation is supported by a recent eye-tracking study showing that altercentric interference is greatest when participants have to switch between their own and the avatar’s perspective across consecutive trials (Ferguson et al., 2017), and a computerized false-belief task showing that switching perspectives from self-to-other is more costly than from other-to-self (Bradford et al., 2015). Finally, Experiment 1 tested a high number of trials (essential for ERP analysis, see Luck, 2014) as in McCleery et al. (2011), which is significantly higher than is typically used in behavioural studies (e.g., Samson et al., 2010, N = 208), and thus may have led to fatigue in our participants. This possibility was tested in a post-hoc analysis on reaction time data, including only the first half of experimental trials, which replicated the finding that age of avatar did not modulate the Perspective × Consistency interaction (*F* = .64, *p* = .43). As such, fatigue is less likely to account for reaction time insensitivity to the predicted avatar-dependent modulation of the perspective effect.

In Experiments 2 and 3, we employed a purely behavioural design (no ERPs), which allowed us to test a larger sample of participants in a within-subjects design, in line with previous research (e.g., Cole et al., 2016; Conway et al., 2017). While employing such a within-subjects design alongside ERPs would be ideal to fully understand the observed ERP effects, the necessary impact on increased trial numbers makes this option unviable (i.e., this design would require 1248 trials in total to match trials per condition (N = 72) to Experiment 1). By not recording ERPs, we were able to significantly reduce the number of trials (Expt. 1 = 624 vs*.* Expt. 2 = 312 vs. Expt. 3 = 208 trials). Thus, in Experiment 2 we tested the effects of age of avatar in a fully crossed within-subjects design that tapped both self and other perspectives to examine whether age of avatar effects would be evident on behavioural responses when effects from individual differences (resulting from Experiment 1’s mixed design) were eliminated. We expected to replicate the egocentric and altercentric effects on accuracy and reaction time measures. More important for the current research, if the effects of avatar seen on P300 and LFSW amplitudes genuinely reflect distinct self-other biases for adult and child observers then we expected to observe this avatar × consistency × perspective interaction on the behavioural responses in Experiment 2. Specifically, we predicted that egocentric interference would disrupt reaction times for both adult and child avatars, but that altercentric interference would only be observed for an adult, and not a child, avatar.

In Experiment 3 we further examined whether age of avatar influences ‘pure’ altercentric intrusion effects by testing self-perspective trials in isolation. Thus, if the altercentric effect truly reflects age-biased differences in spontaneous other perspective-taking then we expected to see reduced altercentric interference for child *versus* adult avatars when participants were never prompted to take the avatars perspective. In contrast, if these effects purely reflect carry-over effects from explicit, non-automatic mentalizing on other perspective trials, then we would expect the age-modulation of the altercentric effect to disappear when the self perspective was assessed in isolation.

## Experiment 2

### Method

#### Participants

Fifty-nine English-speaking Caucasian students from the University of Kent took part in the study. Four participants were removed due to an overall accuracy at or below chance, two participants were removed due to one or more conditions with 0% accuracy, and one participant was removed due to an average response time (*M* = 927 ms) that fell more than 2.5 standard deviations from the mean of all other participants (*M* = 602 ms; *SD* = 99.7). Thus, the final sample consisted of 52 participants (39 female; 50 right-handed; *M*_age_ 19.8 years). Sample size was determined *a priori* to match the target sample size used in Conway et al.’s (2017) Experiment 3 that manipulated three independent variables in a visual perspective-taking task (*N* = 54), and which was three times the size of Furlanetto et al. (2016; N = 18).

#### Materials

The visual stimuli were identical to those used in Experiment 1.

#### Procedure

The procedure was a modified version of Experiment 1, based on that used by Furlanetto et al. (2016) and Conway et al. (Conway et al., 2017, Experiment 3), where participants verified the number of discs according to their own or the avatar’s perspective, and age of avatar was manipulated within participants. Only behavioral responses were recorded.

The task began with a practice block of 26 trials, followed by six blocks of 52 trials (24 matching, 24 mismatching, and four fillers). Three consecutive blocks included an adult avatar in the center of the room, and the next three consecutive blocks included a child avatar; the order of these blocks was counterbalanced across participants. Half the trials (24 per block) were consistent and half were inconsistent. Trials were presented in a pseudorandom order within each block, with the same constraints as in Experiment 1, and the full experiment lasted approximately 40 min.

### Results

**Fig. 8:**
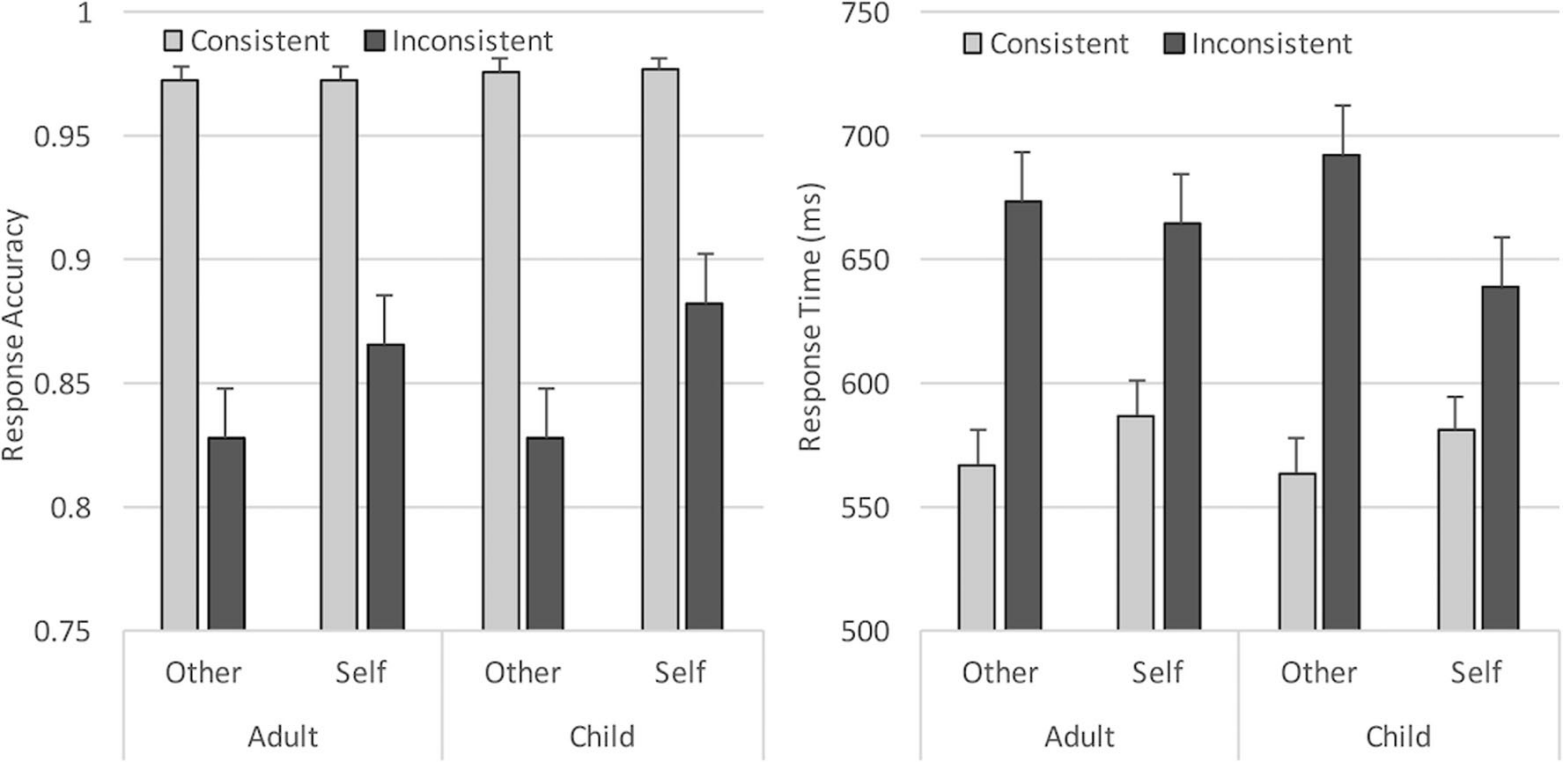
Mean response accuracy and response times for each condition in Experiment 2. Error bars show standard errors

Accuracy and response times for matching trials were analyzed using within-subjects 2 × 2 × 2 × 2 ANOVAs, crossing Perspective (Self vs*.* Other), Consistency (Consistent vs. Inconsistent), Avatar (Adult vs*.* Child), and Order (Adult avatar first vs*.* Child avatar first). Incorrect responses and trials where the participant did not respond to the image in the given 2,000 ms (10%) were excluded from the response-time analysis. Mean response accuracy and response times for each condition are shown in Fig. 8.

#### Response accuracy

Overall accuracy was high (91.3%). The ANOVA revealed the expected significant main effect of Consistency (*F*(1, 50) = 93.23, *p* < .001, _p_η^2^ = .65), showing higher accuracy when participants shared the same visual perspective with the avatar (*M* = 97.5%), compared to when the two perspectives were inconsistent (*M* = 85.1%). In addition, the Perspective × Consistency interaction was significant (*F*(1, 50) = 4.01, *p* = .05, _p_η^2^ = .07). Bonferroni corrected *post hoc* tests revealed a larger consistency effect when taking the other perspective (*t*(51) = 7.98, *p* < .001; consistent *minus* inconsistent = 15%), compared to when taking the self perspective (*t*(51) = 6.52, *p* < .001; consistent *minus* inconsistent = 10%); paired-samples t-test comparing the consistency effect in each perspective condition: *t*(51) = 2.0, *p* = .051. None of the remaining main effects or interactions reached significance (*F*s < 3.36, *p*s > .07).

#### Response times

The ANOVA showed a significant main effect of Consistency (*F*(1, 50) = 105.86, *p* < .001, _p_η^2^ = .68), with slower responses when self and other perspectives were inconsistent (*M* = 668 ms) compared to when they were consistent (*M* = 575 ms). In addition, Perspective interacted significantly with Avatar (*F*(1, 50) = 6.84, *p* = .012, _p_η^2^ = .12). Overall, processing of the self versus other perspective was enhanced when a child avatar was present (*t*(51) = 2.02, *p* = .05; other *minus* self = 19 ms), but there was no difference when an adult avatar was present (*t*(51) = .56, *p* = .58; other *minus* self = -6 ms). However, this effect was further modulated by Order (*F*(1, 50) = 7.57, *p* = .008, _p_η^2^ = .13), which showed that the Perspective × Avatar interaction was only significant when the child avatar condition was tested first (*F*(1, 23) = 14.39, *p* = .001, _p_η^2^ = .39), and not when the adult avatar was tested first (*F*(1, 27) = .01, *p* = .92). Perspective also interacted significantly with Consistency (*F*(1, 50) = 21.44, *p* < .001, _p_η^2^ = .3), showing the expected pattern of greater egocentric interference on other trials (*t*(51) = 11.34, *p* < .001; inconsistent *minus* consistent = 118 ms) than altercentric interference on self trials (*t*(51) = 6.48, *p* < .001; inconsistent *minus* consistent = 68 ms); paired-samples t-test comparing consistency effect in each perspective condition: *t*(51) = 4.65, *p* < .001.

Crucially, the three-way interaction between Avatar, Perspective, and Consistency was significant (*F*(1, 50) = 4.46, *p* = .04, _p_η^2^ = .08), and was further modulated by Order (*F*(1, 50) = 4.04, *p* = .05, _p_η^2^ = .08). Follow-up analyses showed that the Avatar × Perspective × Consistency interaction was only significant when the child avatar condition was tested first (*F*(1, 23) = 6.11, *p* = .02, _p_η^2^ = .21), and not when the adult avatar was tested first (*F*(1, 27) = .01, *p* = .93). The three-way interaction was therefore examined for the child avatar first context, testing effects for adult and child avatars separately. The adult avatar condition showed a significant consistency effect (*F*(1, 23) = 37.14, *p* < .001, _p_η^2^ = .62; consistent < inconsistent), but no Perspective × Consistency interaction (*F*(1, 23) = .13, *p* = .72). In contrast, the child avatar condition revealed significant main effects of Perspective (*F*(1, 23) = 7.96, *p* = .01, _p_η^2^ = .26; self *<* other) and Consistency (*F*(1, 23) = 41.40, *p* < .001, _p_η^2^ = .64; consistent *<* inconsistent), and a significant Perspective × Consistency interaction (*F*(1, 23) = 14.76, *p* < .001, _p_η^2^ = .39). Bonferroni corrected *post hoc* tests revealed that the Consistency effect was significantly larger when taking the Other perspective (*t*(23) = 6.30, *p* < .001; inconsistent *minus* consistent = 158 ms), compared to when taking the Self perspective (*t*(23) = 3.47, *p* = .002; inconsistent *minus* consistent = 57 ms); paired-samples t-test comparing consistency effect in each perspective condition: *t*(23) = 3.16, *p* = .004.

None of the remaining main effects or interactions reached significance (*F*s < 1, *p*s > .4).

#### Summary

In sum, Experiment 2 further replicated egocentric and altercentric interference during visual perspective-taking, with larger egocentric than altercentric effects. Crucially, response time data revealed that the altercentric effect was modulated by age of avatar; interference was significantly weaker for a child versus an adult avatar. This pattern is therefore consistent with the ERP effects on P300 and LFSW in Experiment 1, and thus provides further evidence that participants inferred different mental states for child and adult avatars, possibly due to an own-age bias. Interestingly, this effect only occurred when the child avatar condition was tested first; when the child avatar blocks were preceded by blocks with an adult avatar the consistency effect was comparable for adult and child avatars. This pattern shows that the experimental context has a strong influence on behavioral perspective-taking effects. When perspective was task-relevant, the automatic attentive processing of an adult avatar’s perspective in the first half of the experiment increased the salience of a child avatar’s perspective in subsequent blocks by prompting participants to attend more closely to the avatar’s visual perspective. In line with our predictions, however, automatic processing of the child’s perspective was reduced when attentive processing was not primed by an adult avatar, even when that child avatar’s perspective was task-relevant. In Experiment 3 we examined these context effects further by testing age of avatar effects on self-perspective trials in isolation (i.e., where the avatar’s perspective was task-irrelevant).

## Experiment 3

### Method

#### Participants

Forty-eight English-speaking Caucasian students from the University of Kent took part in the study. Two participants were removed due to an overall accuracy at or below chance, and one participant was removed due to an average response time (*M* = 1156, ms) that fell more than 2.5 standard deviations from the mean of all other participants (*M* = 578 ms; *SD* = 100). Thus, the final sample consisted of 45 participants (39 female; 39 right-handed; *M*_age_ 19.6 years). Sample size was determined *a priori* to match the target sample size used in Conway et al.’s (2017) “cloaked” avatar visual perspective-taking task that tapped the self perspective only (*N* = 48), which was three times the size of Samson et al.’s (2010) original dot perspective task (N = 16).

#### Materials and procedure

The visual stimuli were identical to those used in Experiment 1. The procedure was based on that used by Samson et al. (2010; Experiment 3; see also Cole et al., 2016; Conway et al., 2017; Santiesteban et al., 2014). Participants were asked to verify the number of discs according to their own perspective on every trial; the avatar’s perspective was never probed or mentioned. Only behavioral responses were recorded.

The task began with a practice block of 26 trials, followed by four blocks of 52 trials (24 matching, 24 mismatching, and four fillers). Two consecutive blocks included an adult avatar in the center of the room, and the next two consecutive blocks included a child avatar; the order of these blocks was counterbalanced across participants. Half the trials (24 per block) were consistent and half were inconsistent. Trials were presented in a pseudorandom order within each block, with the same constraints as in Experiment 1, and the full experiment lasted approximately 20 min.

### Results

**Fig. 9:**
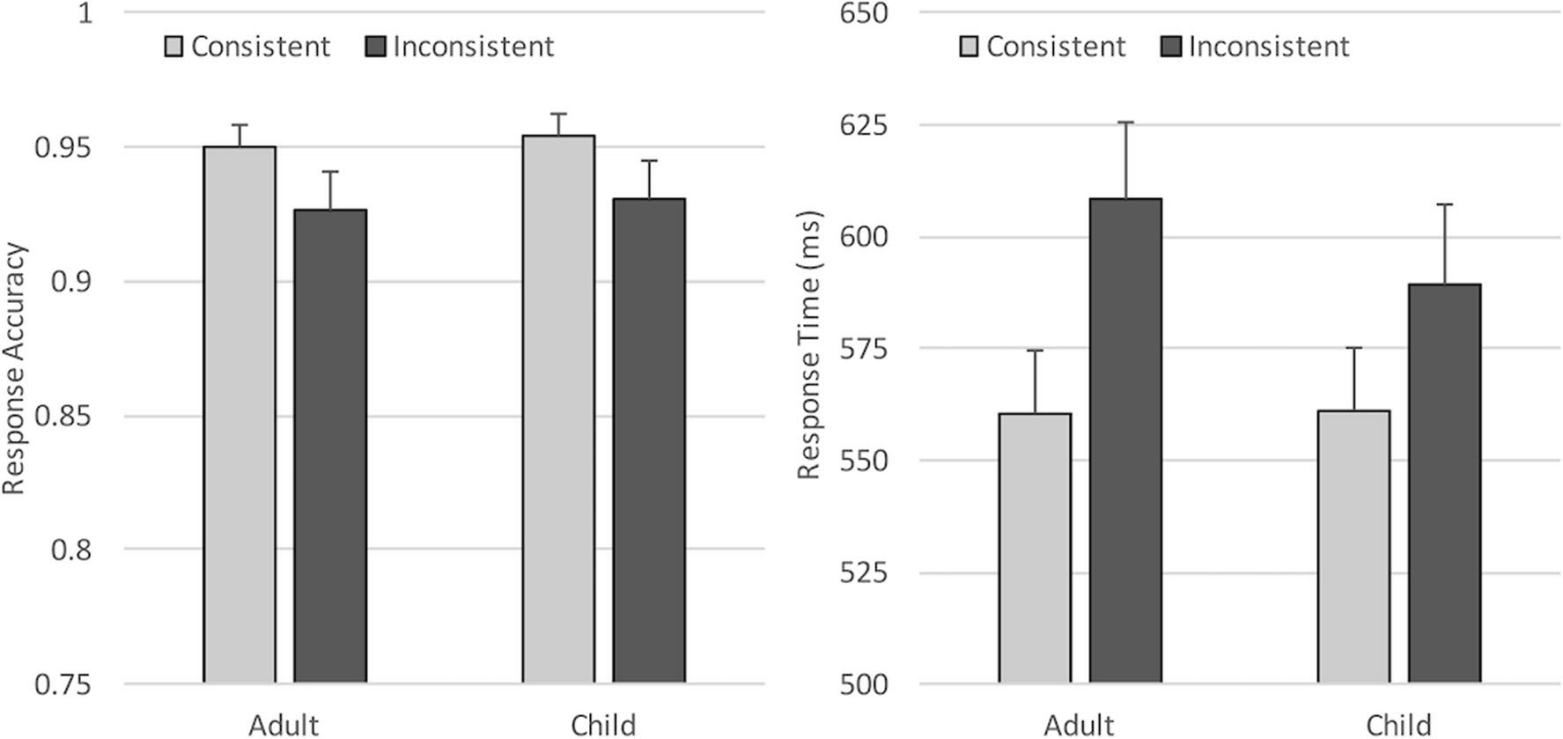
Mean response accuracy and response times for each condition in Experiment 3. Error bars show standard errors

Accuracy and response times for matching trials were analyzed using within-subjects 2 × 2 × 2 ANOVAs, crossing Consistency (Consistent vs. Inconsistent), Avatar (Adult vs*.* Child), and Order (Adult avatar first vs*.* Child avatar first). Incorrect responses and trials where the participant did not respond to the image in the given 2,000 ms (6.3%) were excluded from the response-time analysis. Mean response accuracy and response times for each condition are shown in Fig. 9.

#### Response accuracy

Overall accuracy was high (94%); however, the ANOVA revealed a marginal main effect of Consistency (*F*(1, 43) = 4.00, *p* = .052, _p_η^2^ = .09), showing higher accuracy when participants shared the same visual perspective with the avatar (*M* = 95.2%), compared to when the two perspectives were inconsistent (*M* = 93%). None of the remaining main effects or interactions were significant (*F*s < 1.93, *p*s > .17).

#### Response times

The ANOVA showed a significant main effect of Consistency (*F*(1, 43) = 26.97, *p* < .001, _p_η^2^ = .39), with slower responses when the avatar’s perspective was inconsistent (*M* = 600 ms) compared to consistent (*M* = 561 ms) with the participant’s view. Crucially, this effect of Consistency was modulated by Avatar (*F*(1, 43) = 4.56, *p* = .038, _p_η^2^ = .1), reflecting a larger consistency effect when the central avatar was an adult, *t*(44) = 4.67, *p* < .001 (Inconsistent *minus* Consistent = 48 ms), compared to when the avatar was a child, *t*(44) = 4.03, *p* < 0.001 (Inconsistent *minus* Consistent = 28 ms); paired-samples t-test comparing the consistency effect in each avatar condition: *t*(44) = 2.13, *p* = .04. The main effect of Avatar was not significant (*F* = 1.34, *p* = .25). The Avatar × Block order interaction was marginal, *F*(1, 43) = 3.5, *p* = .068, _p_η^2^ = .08, reflecting faster overall reaction times for child than adult avatars when the adult avatar was tested in the first block (564 vs*.* 586 ms), but no difference between child and adult avatars when the child avatar was tested in the first block (588 vs*.* 582 ms). None of the other effects were close to significance, *F*s < 1.7, *p*s > .2.

#### Summary

Experiment 3 further replicated altercentric effects on visual perspective-taking, even when participants were only ever instructed to refer to their own perspective during the experiment (i.e., the avatar’s perspective was task-irrelevant). Importantly, response times showed that this interference effect was significantly reduced when a child avatar was present compared to when an adult avatar was present. In contrast to Experiment 2, the presentation order of child/adult avatar conditions did not modulate the effect of avatar on consistency, which suggests that an adult avatar only enhances automatic attentive processing of a child’s visual perspective when the experimental context makes this perspective task-relevant.

### General discussion

The experiments presented here examine for the first time how the age of an observed person (adult vs. child avatar) influences adults’ visual perspective-taking, particularly the degree to which they experience interference from their own or the other person’s perspective. In Experiment 1 participants completed the avatar visual perspective-taking task for both self and other perspectives, and age of avatar was manipulated between two groups. We recorded ERPs alongside behavioral measures (reaction time and response accuracy) to examine the effects of perspective-taking and age of avatar in real-time. In Experiments 2 and 3 we replicated the age of avatar manipulation in a within-subjects design, and examined “pure” altercentric intrusion effects by testing self-perspective trials in isolation (Experiment 3), with only behavioral responses recorded.

In line with previous experiments that have employed this task (e.g., Catmur et al., 2016; Conway et al., 2017; Ferguson et al., 2017; Nielsen et al., 2015; Samson et al., 2010; Santiesteban et al., 2014; Qureshi et al., 2010), participants experienced difficulty ignoring the irrelevant perspective (i.e., what they saw and what the avatar saw) when the two perspectives differed. That is, responses were slower and less accurate, and ERPs revealed smaller P200/P300/LFSW amplitudes, when the self and other’s visual perspectives were inconsistent. As in previous studies, egocentric interference was greater than altercentric interference, as one’s own perspective elicited greater disruption to perspective-taking than the avatar’s perspective (on reaction times and P200/P300/LFSW amplitudes).

#### Age of avatar effects on perspective-taking

The most important finding across these three experiments is that age of avatar modulated the magnitude of altercentric interference on self-perspective trials. In Experiment 1, the amplitude of P300 and LFSW ERP components was influenced by the consistency of self and other perspectives for adult avatars only; altercentric effects on these components were eliminated when a child avatar was present. In Experiments 2 and 3, this pattern was corroborated by behavioral data, with reaction times showing a smaller consistency effect for self trials when the central avatar was a child compared to when the avatar was an adult.^4^ These findings are consistent with prior research suggesting that the degree to which people spontaneously infer other peoples’ mental states is influenced by the degree of similarity that they feel with that other person (e.g., Davis et al., 1996; Mahajan & Wynn, 2012; Mitchell et al., 2006; Pfeifer et al., 2009; Smith & Mackie, 2016). Interestingly, age of avatar did not influence the magnitude of egocentric biases. This pattern suggests that although the automatic processing of perspectives is weakened for dissimilar child avatars, observers do not compensate by increasing their reliance on the self perspective. This finding is an interesting contrast to previous studies that have reported greater egocentric interference, but no increase in altercentric interference, when people are taking the perspective of an ingroup member compared to an outgroup member (e.g., Savitsky et al., 2011; Simpson & Todd, 2017; Todd et al., 2011). We attribute this difference to the salience and magnitude of similarity between the self and other between experiments. Specifically, Simpson and Todd (2017) manipulated in/out groups in the avatar visual perspective-taking task using short-term or temporary affiliations. In Experiment 1 the avatar was a University mascot (so affiliations were related to individuals’ length of time at University and interest in University sports), and Experiment 2 used a minimal-group design, where the basis for group membership was arbitrary and temporary (i.e., affiliations were based on “personality colors”). In addition, in Simpson and Todd’s Experiment 1 the University mascot was a bird rather than a human avatar, which may have reduced other perspective-taking effects and increased participants’ reliance on the self perspective. In contrast, the current experiments used realistic images of human avatars in a 3D room, and similarity to the avatar was manipulated based on a salient and long-term property of group membership – age. Clearly both manipulations provide valuable insights into the effect of similarity on self and other perspective judgments, and together the results suggest that egocentric/altercentric tendencies are modulated by the strength of affiliation to in/out groups.

We attribute the reduced altercentric effect in the current experiments to an own-age bias whereby adult participants experienced enhanced processing of the own-age avatar’s perspective. This effect is consistent with prior research that has shown heightened attention towards faces that are in the same age category as the perceiver (e.g., Bailey et al., 2014), superior memory for faces of one’s own age group (Rhodes & Anastasi, 2012), and higher judgments of trust for own-age relative to other-age people (Slessor et al., 2014). Particularly relevant to our study is Slessor et al.’s (2010) research showing that young adults exhibit enhanced eye-gaze following for own-age faces compared to faces of older adults. Although this study did not directly compare effects for adult versus child faces, the findings suggest that young adults preferentially process gaze cues from the faces of their own age group. Gaze direction provides a strong cue in guiding attention towards the location of an actor's gaze (Borji, Parks, & Itti, 2014; Castelhano, Wieth, & Henderson, 2007). Thus, applied to our own results, this implies that the adult avatar’s eye gaze was more salient to our young adult participants than the child avatar’s eye gaze, which increased the likelihood that participants spontaneously inferred the adult’s visual perspective. Further research is needed to validate this attentional account of the age-related altercentric effect in our data, ideally using eye-tracking (as in Ferguson et al., 2017) to examine whether early visual biases to the avatar’s gaze location are reduced for child versus adult avatars.

An alternative explanation for the reduced interference from a child avatar’s perspective is that our adult participants may have assumed a reduced mental capacity for children compared to adults, or placed less importance on their differing perspective due to their young age. Although to our knowledge no research to date has directly tested this suggestion, there is plenty of evidence to suggest that this may be the case. For example, children do not develop all the necessary skills for complex social communication until around 9 years old (Hollebrandse, van Hout, & Hendricks, 2014; Perner & Wimmer, 1985; Sullivan, Zaitchik, & Tager-Flusberg, 1994; Wellman et al., 2001), and in fact ToM continues to develop throughout adolescence and well into our twenties (e.g., Blakemore, 2008). In addition, metacognitive abilities (i.e., the capacity to reflect on one’s own thoughts and behaviours) that are closely related to ToM (Carruthers, 2009; Efklides, 2008; Kuhn, 2000; Schneider, 2008) show a prolonged developmental trajectory, reaching a peak in late adolescence (Weil et al., 2013). Thus, future research should disentangle whether the reduced altercentric intrusion effects seen for child avatars in the current study are driven by a general enhancement of visual processing for own-age others, or a more specific effect that is driven by assumptions of reduced mental awareness in younger children.

#### Distinguishing mentalizing and attention effects during perspective-taking

Our results also make an important contribution to the debate about mentalizing versus directional bases of the altercentric effect in this avatar visual perspective-taking task. The fact that altercentric interference was reduced for the child avatar, even when the avatar’s perspective was task-irrelevant, suggests that adults do not automatically compute the visual perspective of a child avatar, and that altercentric effects for the adult avatar were truly spontaneous and not attributable to simple carry-over effects from other perspective trials (Cole et al., 2016; Conway et al., 2017). To our knowledge, only a mentalizing account would predict differences in perspective-taking based on the age of the avatar (also the in/out group effects reported in Simpson & Todd, 2017), since directional features were matched between avatars (as shown by the Posner attentional pre-test). This finding therefore conflicts with a purely attentional explanation for the altercentric pattern (e.g., Catmur et al., 2016; Cole et al., 2016; Conway et al., 2017; Heyes, 2014; Santiesteban et al., 2014). Nevertheless, we do not rule out the influence of sub-mentalizing on automatic perspective-taking, since directional features of the avatar clearly do provide low-level cues to guide attention (as shown in our pre-test and previous research, e.g., Cole et al., 2016). Instead we propose that *both* implicit mentalizing and directional processes underlie the altercentric effect, as suggested by previous ERP research that has observed modulations of the P300 and LFSW components in different social contexts. Similarity to self may therefore modulate the degree to which observers rely on the self/other perspective via a top-down process that focuses attention onto differences in mental states/capacity or altered gaze following (e.g., Slessor et al., 2010).

The current research is among a growing number of published studies that have examined the brain’s electrophysiological responses during self/other perspective inferences, thus we can begin to interpret the underlying mechanisms within this context. First, the finding that perspective-inconsistent and perspective-consistent conditions were distinguishable on the P300 component supports the proposal that P300 indexes the self-other distinction and conflict resolution in social contexts. Moreover, our results support the proposal that modulations of the P300 reflect social inferences that distinguish between self and other perspectives, as well as the recruitment of higher-order cognitive processes to evaluate self-related stimuli, including increased allocation of attention and conflict resolution. The direction of the consistency effect (i.e., smaller P300 amplitudes for inconsistent vs*.* consistent conditions) replicates that seen in tasks that require self/other conflict monitoring (e.g., Cygan et al., 2014; Deschrijver et al., 2016, 2017), and suggests that P300 amplitude is reduced when interference between perspectives is high, and cognitive resources are required elsewhere (i.e., to manage conflict in inconsistent conditions; Deschrijver et al., 2017). Our results extend this work by showing that this disruption is asymmetric for self/other perspectives – the consistency effect was greater when the conflict comes from one’s own perspective than from someone else’s (reflected in the perspective × consistency interaction on P300 amplitude) – and can be differentially influenced by features of the other person, such as their age (reflected in the age × perspective × consistency interaction on P300 amplitude). In addition, perspective and consistency modulated effects on the LFSW. Previous research has linked this waveform to the processes of distinguishing mental states from reality (Liu et al., 2004; Sabbagh & Taylor, 2000), including the calculation of conflicting self/other perspectives (Jiang et al., 2016), and the recruitment of domain-general executive processes to inhibit the self perspective when inferring others’ perspectives (McCleery et al., 2011; Zhang et al., 2009). We note that perspective and consistency influenced the amplitude of P300 and LFSW in comparable ways, therefore we are unable to distinguish the social and executive processes that underlie each of these components. However, our exploratory comparison of P300/LFSW effects suggests that the consistency effect is dissociable at the scalp level, thus suggesting that the P300 and LFSW might reflect distinct stages of processing. Further research is needed to systematically test these contributions and provide a clearer understanding of the underlying mechanisms and their timecourse.

Our Experiment 1 is one of only two studies that have recorded ERPs while participants complete the avatar visual perspective-taking task (cf. McCleery et al., 2011). Despite differences in the modality of stimulus presentation, there are clear consistencies in the patterns of ERP effects between our experiment (that employed a fully visual design) and McCleery et al. (2011; who used a multi-modal auditory-visual design). Specifically, the P200 was modulated by perspective inconsistencies in both studies, showing sensitivity to the different attentional demands when participants were required to attend to/ignore discs on both walls (i.e., larger P200 peak for the self-inconsistent condition compared to other-inconsistent). Effects on our P300 component were also consistent with McCleery et al.’s TP450, revealing an effect of perspective on peak latencies (other > self), and an effect of consistency on peak amplitude (consistent > inconsistent), and thus suggesting that these components reflect comparable self/other distinction processes. Further, the LFSW amplitude distinguished consistent and inconsistent trials in both experiments; however, the direction of this consistency effect differed between experiments. We attribute these opposing effects on the LFSW to the distinct and non-overlapping time intervals used for calculating mean LFSW amplitudes across experiments. Specifically, our LFSW time window (400–700 ms) was selected to cover processes leading up to the keyboard response (~650 ms), and was therefore likely to include processes that underlie the calculation of self/other perspectives and conflict management. In contrast, McCleery et al. observed effects over a later interval (600–800 ms), which covered processes following the keyboard response, and thus was more likely to reflect inhibitory processes involved in selecting the appropriate perspective than calculating perspectives (since this should have been complete). Nevertheless, these findings highlight the value of measuring implicit and explicit perspective-taking in the same task, since ERPs revealed implicit sensitivity to the avatar’s perspective even before participants had made an explicit response. The value of this electrophysiological approach is further demonstrated in Experiment 1 since the age of avatar effects on the P300 and LFSW components were not visible on the explicit behavioral response measures. Therefore, future work that examines visual perspective-taking would benefit from examining the time-course of implicit neural processing in this way.

### Conclusion

The experiments reported here provide a novel extension to research that has examined visual perspective-taking in a healthy adult population. Across three experiments that use behavioral and ERP measures we show the first evidence that the degree to which people experience interference from another’s (conflicting) visual perspective is modulated by the age of that other person; children elicit significantly reduced altercentric interference compared to adults. Moreover, the degree to which perspective-taking was disrupted for a child avatar was strongly influenced by constraints from the experimental context. We attribute this effect to an own-age bias, whereby adult participants experienced enhanced processing of the own-age avatar’s perspective, which could reflect either enhanced visual processing for own-age others or an inference on reduced mental awareness in younger children. These findings argue against the suggestion that the altercentric effect reflects purely attentional features of the avatar, and instead support an account where *both* mentalizing and directional processes modulate automatic visual perspective-taking.

## Appendix

**Table 1:** Statistical effects for each behavioral measure in Experiment 1. Asterisks show significance of effects, where * *p* < 0.05; ** *p* < 0.01; *** *p* < 0.001

		df	*F*	*p*	_p_η^2^
Response accuracy	Perspective	1, 32	4.2	.05 *	.12
	Consistency	1, 32	72.19	<.001 ***	.69
	Avatar	1, 32	.20	.66	<.01
	Perspective × Avatar	1, 32	2.25	.14	.07
	Consistency × Avatar	1, 32	.02	.88	<.01
	Perspective × Consistency	1, 32	1.73	.2	.05
	Perspective × Consistency × Avatar	1, 32	.66	.42	.02
Response times	Perspective	1, 32	1.72	.20	.05
	Consistency	1, 32	93.62	<.001 ***	.75
	Avatar	1, 32	.17	.69	<.01
	Perspective × Avatar	1, 32	.69	.41	.02
	Consistency × Avatar	1, 32	.20	.66	<.01
	Perspective × Consistency	1, 32	16.65	<.001 ***	.34
	Perspective × Consistency × Avatar	1, 32	.54	.47	.02

**Table 2:** Statistical effects for each ERP measure in Experiment 1. Asterisks show significance of effects, where * *p* < 0.05; ** *p* < 0.01; *** *p* < 0.001

		df	*F*	*p*	_p_η^2^
P100 amplitude	Perspective	1, 32	1.16	.29	.04
	Consistency	1, 32	2.82	.1	.08
	Avatar	1, 32	4.73	.04 *	.13
	Perspective × Avatar	1, 32	2.0	.17	.06
	Consistency × Avatar	1, 32	.21	.65	<.01
	Perspective × Consistency	1, 32	<.01	.98	<.01
	Perspective × Consistency × Avatar	1, 32	1.76	.19	.05
P200 amplitude	Perspective	1, 32	9.99	.003 **	.24
	Consistency	1, 32	27.66	<.001 ***	.46
	Avatar	1, 32	1.51	.23	.05
	Perspective × Avatar	1, 32	.92	.34	.03
	Consistency × Avatar	1, 32	1.29	.26	.04
	Perspective × Consistency	1, 32	7.83	.009 **	.2
	Perspective × Consistency × Avatar	1, 32	3.07	.09	.09
P300 latency	Perspective	1, 32	25.26	<.001 ***	.45
	Consistency	1, 32	2.57	.12	.07
	Avatar	1, 32	.29	.59	<.01
	Perspective × Avatar	1, 32	.08	.78	<.01
	Consistency × Avatar	1, 32	.2	.66	<.01
	Perspective × Consistency	1, 32	.13	.72	<.01
	Perspective × Consistency × Avatar	1, 32	.38	.54	.01
P300 amplitude	Perspective	1, 32	3.01	.09	.09
	Consistency	1, 32	104.63	<.001 ***	.77
	Avatar	1, 32	.6	.44	.02
	Perspective × Avatar	1, 32	4.8	.04 *	.13
	Consistency × Avatar	1, 32	.21	.65	<.01
	Perspective × Consistency	1, 32	.64	.43	.02
	Perspective × Consistency × Avatar	1, 32	5.41	.03 *	.15
LFSW	Perspective	1, 32	6.28	.02 *	.16
	Consistency	1, 32	8.90	.005 ***	.22
	Avatar	1, 32	.04	.84	<.01
	Hemisphere	1, 32	5.02	.03 *	.14
	Perspective × Avatar	1, 32	3.36	.08	.10
	Consistency × Avatar	1, 32	.74	.40	.02
	Perspective × Consistency	1, 32	.42	.52	.01
	Perspective × Hemisphere	1, 32	.09	.77	<.01
	Consistency × Hemisphere	1, 32	.10	.76	<.01
	Hemisphere × Avatar	1, 32	.18	.67	<.01
	Perspective × Consistency × Avatar	1, 32	7.45	.01 **	.19
	Perspective × Consistency × Hemisphere	1, 32	.001	.96	<.01
	Perspective × Hemisphere × Avatar	1, 32	3.01	.09	.09
	Consistency × Hemisphere × Avatar	1, 32	.01	.92	<.01
	Perspective × Consistency × Hemisphere × Avatar	1, 32	1.47	.24	.04

**Table 3:** Statistical effects for each behavioral measure in Experiment 2. Asterisks show significance of effects, where * *p* < 0.05; ** *p* < 0.01; *** *p* < 0.001

		df	*F*	*p*	_p_η^2^
Response accuracy	Perspective	1, 50	3.36	.07	.06
	Consistency	1, 50	93.23	<.001 ***	.65
	Avatar	1, 50	.75	.39	.02
	Order	1, 50	.13	.72	<.01
	Perspective × Avatar	1, 50	.22	.64	<.01
	Consistency × Avatar	1, 50	.14	.71	<.01
	Order × Avatar	1,50	<.01	.99	<.01
	Perspective × Consistency	1, 50	4.01	.05 *	.07
	Perspective × Order	1, 50	.64	.43	.01
	Consistency × Order	1, 50	.15	.71	<.01
	Perspective × Consistency × Avatar	1, 50	.27	.61	.01
	Perspective × Consistency × Order	1, 50	<.01	.99	<.01
	Perspective × Avatar × Order	1, 50	.03	.87	<.01
	Consistency × Avatar × Order	1, 50	.02	.90	<.01
	Perspective × Consistency × Avatar × Order	1, 50	.02	.89	<.01
Response times	Perspective	1, 50	.70	.41	.01
	Consistency	1, 50	105.86	<.001 ***	.68
	Avatar	1, 50	.02	.89	<.01
	Order	1, 50	.69	.41	.01
	Perspective × Avatar	1, 50	6.84	.01 *	.12
	Consistency × Avatar	1, 50	.05	.83	<.01
	Order × Avatar	1,50	10.45	.002 **	.17
	Perspective × Consistency	1, 50	21.44	<.001 ***	.3
	Perspective × Order	1, 50	.04	.85	<.01
	Consistency × Order	1, 50	.19	.66	<.01
	Perspective × Consistency × Avatar	1, 50	4.46	.04 *	.07
	Perspective × Consistency × Order	1, 50	.17	.69	<.01
	Perspective × Avatar × Order	1, 50	7.57	.008 **	.13
	Consistency × Avatar × Order	1, 50	2.31	.14	.04
	Perspective × Consistency × Avatar × Order	1, 50	4.04	.05 *	.08

**Table 4:** Statistical effects for each behavioral measure in Experiment 3. Asterisks show significance of effects, where * *p* < 0.05; ** *p* < 0.01; *** *p* < 0.001

		df	*F*	*p*	_p_η^2^
Response accuracy	Consistency	1, 43	4.00	.05 *	.04
	Avatar	1, 43	.30	.59	<.01
	Order	1, 43	1.93	.17	.04
	Consistency × Avatar	1, 43	.06	.94	<.01
	Consistency × Order	1, 43	.69	.41	.02
	Avatar × Order	1, 43	1.71	.20	.04
	Consistency × Avatar × Order	1, 43	.96	.33	.02
Response times	Consistency	1, 43	26.97	<.001 ***	.39
	Avatar	1, 43	1.13	.30	.03
	Order	1, 43	.11	.74	<.01
	Consistency × Avatar	1, 43	4.56	.04 *	.10
	Consistency × Order	1, 43	1.66	.20	.04
	Avatar × Order	1, 43	3.50	.07	.08
	Consistency × Avatar × Order	1, 43	.22	.64	.01

## Footnotes

1 These age classifications were confirmed by participants at the end of the task. Adult-like avatars were judged to have a median age of 25 years old (Female = 25.3 years old; Male = 24.6 years old), and child-like avatars a median age of 6 years old (Girl = 6.1 years old; Boy = 6.6 years old).

2 Note that these variable ISIs were used to match those used in McCleery et al. (2011), and to eliminate effects of a predictable rhythmic presentation rate on attention and ERP components (see Doherty, Rao, Mesulam, & Nobre, 2005).

3 Anterior and posterior electrode sites were based on those used for the main P300/LFSW analyses, with the anterior site including activity over left and right frontal electrodes (AF7, F7, F5, AF8, F6, F8) and the posterior site including activity over central parietal electrodes (CP1, CP2, CPz, Pz, P1, P2).

4 We attribute the non-significant age of avatar effect on behavioral data in Experiment 1 to the between-groups design, which is likely to have increased noise on this variable.
